# Molecular Insights Into Binding and Activation of the Human KCNQ2 Channel by Retigabine

**DOI:** 10.3389/fmolb.2022.839249

**Published:** 2022-03-03

**Authors:** Barbara Garofalo, Alexandre M.J.J. Bonvin, Andrea Bosin, Francesco P. Di Giorgio, Rosella Ombrato, Attilio V. Vargiu

**Affiliations:** ^1^ Angelini Pharma S.p.A., Rome, Italy; ^2^ Faculty of Science—Chemistry, Bijvoet Center for Biomolecular Research, Utrecht University, Utrecht, Netherlands; ^3^ Department of Physics, University of Cagliari, Cagliari, Italy

**Keywords:** voltage-gated potassium channels, Kv7.2, retigabine, homology modelling, docking, molecular dynamics

## Abstract

Voltage-gated potassium channels of the Kv7.x family are involved in a plethora of biological processes across many tissues in animals, and their misfunctioning could lead to several pathologies ranging from diseases caused by neuronal hyperexcitability, such as epilepsy, or traumatic injuries and painful diabetic neuropathy to autoimmune disorders. Among the members of this family, the Kv7.2 channel can form hetero-tetramers together with Kv7.3, forming the so-called M-channels, which are primary regulators of intrinsic electrical properties of neurons and of their responsiveness to synaptic inputs. Here, prompted by the similarity between the M-current and that in Kv7.2 alone, we perform a computational-based characterization of this channel in its different conformational states and in complex with the modulator retigabine. After validation of the structural models of the channel by comparison with experimental data, we investigate the effect of retigabine binding on the two extreme states of Kv7.2 (resting-closed and activated-open). Our results suggest that binding, so far structurally characterized only in the intermediate activated-closed state, is possible also in the other two functional states. Moreover, we show that some effects of this binding, such as increased flexibility of voltage sensing domains and propensity of the pore for open conformations, are virtually independent on the conformational state of the protein. Overall, our results provide new structural and dynamic insights into the functioning and the modulation of Kv7.2 and related channels.

## Introduction

Potassium channels (K^+^ channels) are primary regulators of intrinsic electrical properties of neurons and their responsiveness to synaptic inputs (Ashcroft, 2000; Jentsch, 2000; Miller, 2000; Hille, 2001). An increase in the membrane conductance to K^+^ ions causes neuronal hyperpolarization and, in most cases, reduces firing frequency, exerting a strong inhibitory function on neuronal excitability. On the other hand, reduction in conductance seems to be a hallmark of the hyperexcitability seen in many pain syndromes ranging from traumatic injuries and painful diabetic neuropathy to autoimmune disorders (Shieh et al., 2000; Villa and Combi, 2016; Nikitin and Vinogradova, 2021).

The Kv7.x subfamily of voltage-gated K^+^ channels, encoded by the KCNQ gene family, consists of five members (Kv7.1—Kv7.5 or KCNQ1–KCNQ5), each showing distinct tissue distribution and subcellular localization, as well as biophysical, pharmacological, and pathophysiological properties (Jentsch, 2000; Robbins, 2001; Brown and Passmore, 2009; Barrese et al., 2018; Abd-Elsayed et al., 2019; Jepps et al., 2021). All these channels assemble into membrane-embedded tetramers. Heteromeric assembly of KCNQ2 and KCNQ3, originally termed the “M-channels”, are primary regulators of intrinsic electrical properties of neurons and of their responsiveness to synaptic inputs (Jentsch, 2000; Robbins, 2001). The M-type potassium current is a slowly activating, non-inactivating voltage-gated current, which occurs at subthreshold potentials. It is named after the proposed pathway of its inhibition, i.e., activation of the muscarinic (M) acetylcholine receptor, which leads to the closure of the channel (Brown and Adams, 1980; Marrion, 1997; Delmas and Brown, 2005; Brown and Passmore, 2009; Hernandez et al., 2009). M-currents contribute to the afterhyperpolarization of the action potentials, the spike frequency adaptation, the shaping of the action potential firing properties, the setting of the resting membrane potential and the regulation of presynaptic functions (Bordas et al., 2015). Studies have shown that the current detected across homo-tetrameric KCNQ2 channels is very similar to the M-current, while KCNQ3 channels are rarely able to generate recordable currents (Maljevic et al., 2003).

**FIGURE 1:**
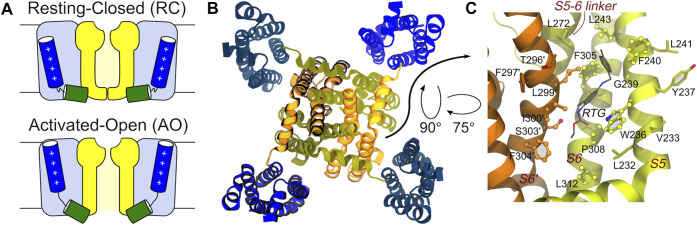
Main conformational states, overall tetrameric structure, and RTG binding site of the KCNQ2 channel. **(A)** Schematic view of the RC and AO conformational states of the channel. The Pore and VSD domains are colored yellow and blue, respectively. The S4 and S4-S5 linker helices are shown as blue and green cylinders, respectively. **(B)** Top view of the tetrameric channel in the AC conformational state (PBD ID: 7CR0). **(C)** Zoom on the RTG binding site on the Pore domain of the KCNQ2 channel. The site is a crevice between two adjacent channel monomers (shown as orange and greenish yellow ribbons). Residues in contact with the drug in the experimental structure 7CR2 (Li et al., 2021) are shown using a CPK model with bright surfaces, while residues identified in this work based on previous knowledge are shown as sticks. Residues on the second monomer are labelled by primed numbers.

KCNQ2 shows a topological arrangement with six transmembrane segments (S1–S6), and intracellular NH2 and COOH termini. The region encompassing segments S1–S4 forms the voltage sensing domain (VSD), with positively charged residues in S4 comprising the main voltage sensor. The S5–S6 region forms the pore domain that contains a P-loop that imparts K^+^ ion selectivity; the carboxylic tail contains four helices (A-D), which contain regions important for tetramerization or binding to regulatory factors including phosphatidylinositol-4,5-biphosphate (PIP2), ankyrin G, and Calmodulin CaM (Soldovieri et al., 2011). The central pore domain is surrounded by four voltage-sensing domains that respond to membrane depolarization by undergoing a conformational change. This in turn triggers structural rearrangements in the pore domain via electromechanical coupling, ultimately opening the channel gate to allow ion conduction (Bezanilla and Stefani, 1994; Jensen et al., 2012). VSD activation occurs stepwise due to the depolarization and proceeds from an initial resting VSD conformation in which the pore is closed (resting/closed, RC) to an activated VSD with an open pore (activated/open, AO) (Bezanilla, 2000; Wu et al., 2010), passing through a conformational change of the VSD into an activated conformation and the pore still closed (activated/closed, AC) as caught by cryo-electron microscopy (cryo-EM) solved structure of KCNQ2 (PDB ID: 7CR0) (Li et al., 2021) (Figure 1).

Functional studies in heterologous expression systems revealed that mutations in KCNQ2 (and KCNQ3) genes are related to the onset of diseases such as epilepsy, benign familial neonatal convulsions (BFNC) or neonatal epileptic encephalopathy (Miceli et al., 2013), peripheral nerve hyperexcitability (PNH or myokymia or neuromyotonia) (Maljevic et al., 2008), neuropathic pain (Di Cesare Mannelli et al., 2017), osteoarthritis or cancer pain, migraine, anxiety, dystonia and dyskinesia, bipolar disorder, bladder hypersensitive disorder, addiction, sensory deficits, stroke (Miceli et al., 2008; Zwierzyńska et al., 2017; Du et al., 2018), mania (Kristensen et al., 2012) and tinnitus (Li et al., 2013). These channels were early identified as pharmacological targets, and M-channel enhancers such as retigabine (hereafter RTG) were developed and approved in humans [with indications and antiepileptic drug (Brown and Passmore, 2009)].

Unfortunately, RTG was withdrawn from the market due to safety issues associated with retina and skin discoloration (Clark et al., 2015). Nonetheless, this compound remains an excellent tool to investigate the mechanism of action related to KCNQ2-opener activity, which is not yet fully unveiled. In particular, while studies have shown that RTG can bind to the hydrophobic pocket located near the channel gate of the subunit of KCNQ2, thereby stabilizing the open conformation of the channel (Gunthorpe et al., 2012), Wang et al. recently suggested that the interaction with KCNQ2 is not gated by channel opening and closing, and that RTG could also interact with the resting states of the protein (Wang et al., 2018).

The binding mode of RTG onto KCNQ2 (featuring an activated VSD and a closed pore—AC) was confirmed by the determination of a cryo-EM structure (Li et al., 2021). The drug sits in a hydrophobic pocket formed by S5, pore helix, and S6 from the neighbouring subunit at the inter-subunit interface in the pore domain (Figure 1). The drug binds to KCNQ2 mainly by hydrogen bonds with the side chain of Trp236, Ser303, and the main chain carbonyls of Leu299, Phe305, as well as the hydrophobic interactions with residues Trp236, Phe240, Leu243, Leu272, Leu299, Phe304 and Phe305. To date there are no KCNQ2 structures available in the RC and AO states and when we performed this work the cryo-EM structure of AC KCNQ2, alone and in complex with RTG (Li et al., 2021), had not yet been published. For this reason, to investigate the structural features underlying the three different conformations, we resorted to using atomistic molecular dynamics (MD) simulations. Here, we report the development and structural validation of new homology models of the human KCNQ2 channel in the RC, AC and AO states. The models turned out to be highly accurate, thus allowing to investigate not only the structural and dynamical features of the channel, but also the binding of RTG to different pore states. By performing ensemble docking and multi-replica MD simulations of the RTG-KCNQ2 complexes in the RC and AO states, we show that this ligand can form stable interactions at the same channel site in both states. Moreover, we reproduce several experimental findings and trends underlying the molecular mechanism behind RTG action, such as a higher flexibility of the VSD domains and an increased tendency to pore opening upon ligand binding.

Overall, our work confirms the high potential of computational methods in this field (Khalili-Araghi et al., 2010; Jensen et al., 2012; Yarov-Yarovoy et al., 2012; Kim et al., 2015; Kasimova et al., 2018; DeMarco et al., 2019; Kuenze et al., 2019; Alberini et al., 2021; Şterbuleac 2021) and provides new insightful data that might help drug design efforts.

## Methods

### Homology Modelling and Validation

We aimed to model the AO, AC, and RC states of the KCNQ2 tetramer. The sequence of KCNQ2 was taken from the UNIPROT website (identifying code: O43526), and the modelling was restricted to the transmembrane region of the channel (residue ARG75 to GLN323 in each monomer), amounting to 249 aminoacids per monomer and including helices S0 to S6. The models were generated using the Prime tool (Jacobson et al., 2004) of the Maestro software package (Prime, Schrödinger, LLC, New York, NY, 2019). Namely, the AC model was generated using as (single) template structure the cryo-EM structure of the homologous KCNQ1 channel (PDB ID: 5VMS) (Sun and MacKinnon, 2017), sharing 58% identity and 75% homology with the sequence of KCNQ2 (SupplementaryTable S1). The sequence alignment was performed with Clustal Omega 1.2.4 (https://www.ebi.ac.uk/Tools/msa/clustalo/). Regarding the AO and RC states, as no experimental structure of KCNQ2 in these conformations was available at the time of this investigation, we used as templates the corresponding structures of the KCNQ1 channel previously published by Kuenze et al. (2019). Validation of the structural models was performed by submitting them to the MOLPROBITY webserver (http://molprobity.biochem.duke.edu/) (Williams et al., 2018).

### Molecular Dynamics Simulations

*Apo proteins.* MD simulations of the AO, RC, and AC models of KCNQ2 in explicit phospholipid membranes at 310 K were performed using Amber18 (Case et al., 2018) and the Lipid17 force field (Gould, I.R., Skjevik A.A., Dickson, C.J., Madej, B.D., Walker, R.C.,"Lipid17: A Comprehensive AMBER Force Field for the Simulation of Zwitterionic and Anionic Lipids”, 2018, in preparation). We employed a protocol similar to that recently used by Kuenze et al. to simulate the transmembrane region of the tetrameric KCNQ1 channel (Kuenze et al., 2019). Namely, the models generated by Prime were used as starting conformations for MD simulations after alignment to the membrane normal using the PPM webserver (Lomize et al., 2012) and embedded into bilayers of POPC (palmitoyloleoyl-phosphatidylcholine; ∼250 phospholipids per leaflet) using the membrane builder tool of the CHARMM-GUI website (Wu et al., 2014). A TIP3P water layer ∼30 Å thick and containing 0.15 M of KCl was added on each side of the membrane. In addition, four K^+^ ions were placed in the channel selectivity filter at positions inferred from the X-ray structure coordinates of PDB 2R9R. The bilayer contained 26 PIP2 (phosphatidyl-4,5-bisphosphate) molecules in the inner leaflet, consisting of an equal number of C4-PO4^-^ and C5-PO4^-^ mono-protonated PIP2 molecules with stearoyl and arachidonoyl conjugations at the sn-1 and sn-2 position. The PIP2 parameters were taken from (Kuenze et al., 2019). No PIP2 was added to the MD system of the AC model because the decoupled state seen in the cryo-EM structure of KCNQ1 is assumed to be due to the absence of PIP2 and the inability of that membrane composition to induce conformational changes. SHAKE bond length constraints were applied to all bonds involving hydrogen. Nonbonded interactions were evaluated with a 10 Å cut-off, and electrostatic interactions were calculated by the particle-mesh Ewald method. Each MD system was first minimized for 15.000 steps using steepest descent for the first 100 steps, followed by conjugate gradient minimization. With protein and ions restrained to their initial coordinates, the lipid and water were heated to 50 K over 1,000 steps with a step size of 1 fs in the NVT ensemble using Langevin dynamics with a rapid collision frequency of 10 ns^−1^. The system was then heated to 100 K over 50.000 steps with a collision frequency of 1,000 ps^−1^ and finally to 310 K over 200.000 steps and a collision frequency of 100 ps^−1^. After switching to the NPT ensemble, restraints on ions were gradually removed over 500 ps and the system was equilibrated for another 25 ns at 310 K with weak positional restraints (force constant of 1 kcal mol^−1^ Å^−2^) applied to protein C_α_ atoms. The protein restraints were then gradually removed over 50 ns, and 9 replicas of production MD, each of 100 ns in length, were conducted for each model (yielding 1 μs for each state) using a step size of 2 fs, constant pressure periodic boundary conditions, anisotropic pressure scaling and Langevin dynamics. Note that, as in (Kuenze et al., 2019), production runs were performed in the presence of soft restraints (k = 2 kcal mol^−1^ Å^−2^) between each K^+^ ion and the 8, 4, 4, and 4 oxygen atoms of residues T277, I278, G279 and Y280 respectively. On top of these runs, for each model and each replica we performed 100 ns additional MD simulations in the absence of restraints. Therefore, for each state we generated a production trajectory corresponding to ∼2 μs of simulated time.

*RTG-KCNQ2 complexes.* Multicopy simulations of the RTG-KCNQ2 complexes derived from docking calculations (*vide infra*) were performed using the same settings as for the simulations of the unbound channel. Force field parameters of the ligand were derived from the GAFF (Wang et al., 2004) force field or generated using the antechamber module of AMBER when missing. In particular, atomic restrained electrostatic potential charges were derived after a structural optimization performed with Gaussian09 (Frisch et al., 2016). For complexes in both the AO and RC conformations, the top eight unique (that is, non-identical) docking poses (selected by visual inspection) were selected as starting conformations for MD simulations. Initial structures of the complexes in a model membrane and water solution were generated by superposing these structures to the conformation extracted the MD trajectory of the apo KCNQ2 channel and corresponding to the cluster representative used in docking calculations. Next, three independent MD simulations, each of 1 μs in length, were performed for each complex structure, amounting to 24 replicas for each system and 48 μs of cumulative time.

### Molecular Docking of RTG on AO and RC States of KCNQ2

In order to assess the possibility of RTG binding to the AO and RC states of the potassium channel KCNQ2, ensemble docking calculations were performed using Autodock4.2 (Morris et al., 2009). Namely, a cluster analysis was performed on the equilibrium trajectories extracted from the cumulative MD simulations of the apo protein, using as a metric the distance RMSD matrix (dRMSD) calculated on the non-hydrogenous atoms of the putative RTG binding site (that is the enlarged binding site in Figure 1) and a 2 Å cut-off. The analysis was performed separately on each of these four binding sites present on the KCNQ2 tetramer and resulted in an overall number of 42 and 17 clusters for AO an RC states, respectively. The default settings of Autodock were used, except for the grid space, which was set to 0.25 (default 0.375), and for the grid volume, which was automatically set so as to cover all residues lining the binding site, following the automated protocol described in (Basciu et al., 2019b). 7 out of the 8 rotatable bonds of RTG were activated during docking; up to 10 poses per channel structure were saved, resulting in 390 poses onto the AO state and 151 onto the RC state.

### Analysis of the MD Trajectories

*Stability of the models.* The stability of our MD simulations was evaluated in terms of the RMSD of the heavy atoms of the protein along the trajectory with respect to the initial structure (i.e., the homology model) as well as to the experimental structures of the KCNQ2 channel in the AC state, as well as of its complex with RTG [PDB IDs 7CR0 and 7CR2, respectively (Li et al., 2021)].

*Root Mean Square Fluctuations (RMSF)*. The flexibility of the protein was evaluated in terms of RMSF calculations, performed on each trajectory with the *atomicfluct* command of the AmberTools. The fluctuations were evaluated after alignment of the Pore domain to the average conformation extracted from the corresponding MD trajectory.

*Identification of the putative binding site of retigabine.* Since no experimental structure of RTG in complex with KCNQ2 was available when this investigation was performed, the putative (lately largely confirmed, see Results) binding site of RTG on the Pore-forming domain of the channels was identified based on the findings in (Lange et al., 2009; Kim et al., 2015). In KCNQ2, the residue W236 was shown to be crucial for the interaction of the channel with retigabine and its analogues (Kim et al., 2015). In particular, the presence of an H-bond donor in this residue was shown to be crucial for binding. Therefore, we identified as putative binding site of retigabine on KCNQ2 the crevice formed by W236 and all the residues within 3.5 Å from it (Figure 1).

*Cluster analysis.* To perform some of the analyses described below within a reasonable time, and to select a tractable number of (maximally different) structures for molecular docking, a cluster analysis was performed on the cumulative trajectory generated by concatenating the 10 independent trajectories generated for each state. We performed two different cluster analyses, using either the C_α_ atoms of the protein or all heavy atoms of the residues around each of the four putative binding sites of retigabine. In this way, we obtained structures differing both in their overall architecture and as well as in the conformations of the putative binding site of retigabine. As metric, we used the dRMSD calculated over the selections above (which were also used for structural alignment), with cut off set to 2 Å. These structural clusters were further analysed as described below.

*Volume and druggability calculations.* Druggability calculations were performed using the *f-pocket* (Le Guilloux et al., 2009) software as described in previous publications (Basciu et al., 2019b; Basciu et al., 2019a). For each conformation of the protein extracted from the cluster analysis described above, we evaluated its druggability score D, a descriptor ranging from 0 to 1 with higher values identifying more druggable geometries (Schmidtke and Barril, 2010). It is customary to associate scores >0.5 to putative binding sites. We also estimated the approximate volume of the retigabine binding sites by using the software VOIDOO (Kleywegt and Jones, 1994) with the following settings: grid spacing 0.5 Å; probe radius 1.4 Å, growth factor for van der Waals radii 1.05, number of cavity refinement cycles 100. The coordinates of the centres of the cavity were set to the geometrical centre of the sites.

*Interaction network.* The intra- and inter-molecular interactions involving the retigabine binding sites were calculated using the *cpptraj* tool of the AMBER18 package (Case et al., 2018, p. 18) and the PLATON software package (Spek, 2009). Namely, the first software was used to characterize the network of H-bonds (given the importance of W236 as H-bond donor), using a cut-off of 3.5 Å for the donor-acceptor distance and of 145° for the donor-hydrogen-acceptor angle. The second software was used to detect stacking interactions, given that a change in the network of residues involved in π-π interactions was suggested to occur upon switch of the channel from a closed to an open state (Syeda et al., 2016). The default parameters were used to detect interactions involving aromatic rings.

*Pore morphology.* For each simulated system, the presence of—and the morphology of putative tunnels leading from the cytoplasmic entrance to the center of the protein (beneath the selectivity filter) were evaluated using CAVER3.0 (Chovancova et al., 2012) on representative structures extracted from the cluster analysis performed on the C_α_ atoms of the whole protein, as described above. The probe radius, shell radius, shell depth, and clustering threshold were set to 0.9 Å, 5.0 Å, 3.0 Å, and 3.5 Å, respectively. Tunnel calculations were started for each conformation at the center between residues 276 and 306 on each monomer.

*Binding free energy estimation*. Free energies of binding for RTG-KCNQ2 complexes were estimated using the molecular mechanics generalized Born surface area (MM-GBSA) approach (Genheden and Ryde, 2015) through the formula:

$$\Delta G_{bind}\;=\;G_{com}\;-\;\left(G_{rec\;}+\;G_{lig}\right)$$

*G*_*com*_, *G*_*rec*_, and *G*_*lig*_ are the absolute free energies of the complex, receptor, and ligand, respectively, averaged over the equilibrium trajectory. According to these schemes, the free energy difference can be decomposed as:

$$\Delta G_{bind}\;=\;\Delta E_{MM}\;+\;\Delta G_{solv}\;-\;T\Delta S_{conf}$$

where *ΔE*_*MM*_ is the difference in the molecular mechanics energy, *ΔG*_*solv*_ is the solvation free energy, and *TΔS*_*conf*_ is the conformational entropy. The first two terms were calculated with the following equations:

$$\Delta E_{MM}\;=\;\Delta E_{bond}\;+\;\Delta E_{angle}\;+\;\Delta E_{torsion}\;+\;\Delta E_{vdw}\;+\;\Delta E_{elec}$$

$$\Delta G_{solv}\;=\;\Delta G_{solv,p}\;+\;\Delta G_{solv,np}$$

*E*_*MM*_ includes the molecular mechanics energy contributed by the bonded (*E*_*bond*_, *E*_*angle*_, and *E*_*torsion*_) and non-bonded (*E*_*vdw*_ and *E*_*ele*_, calculated with no cutoff) terms of the force field. As customary, we employed the single-trajectory approach, in which only simulations of the complex are employed to generate the ensemble of conformations of the receptor and of the ligand by simply removing the appropriate atoms (Genheden and Ryde, 2015). This corresponds to setting $\Delta E_{bond}$ , $\Delta E_{angle}$ , and $\Delta E_{torsion}$ to zero. Δ*G*_*solv*_ is the solvation free energy, which can be modeled as the sum of an electrostatic contribution (Δ*G*_*solv,p*_, evaluated using the MM-GBSA or MM-PBSA approach) and a non-polar one (Δ*G*_*solv,np*_ = *γΔ*_*SA*_ *+ b*, proportional to the difference in solvent-exposed surface area, Δ_*SA*_).

The electrostatic solvation free energy was calculated using the implicit solvent model developed by Nguyen et al. (Nguyen et al., 2013) (igb = 8 option in AMBER) in combination with mbondi2 (for H, C, N, O, S elements) and intrinsic radii. Partial charges were taken from the AMBER/GAFF force fields, and relative dielectric constants of 1 for solute and 78.4 for the solvent were used. The non-polar contribution is approximated by the LCPO method (Weiser et al., 1999).

The solute conformational entropy contribution (TΔS_conf_) was not evaluated as it is notoriously difficult to evaluate with accuracy (Genheden and Ryde, 2015). The estimates were performed on up to 100 different equally spaced conformations extracted from each of the ten most populated clusters calculated from the cumulative trajectories of AO-RTG and RC-RTG complexes.

## Results and Discussion

### Model Building and Validation

**FIGURE 2:**
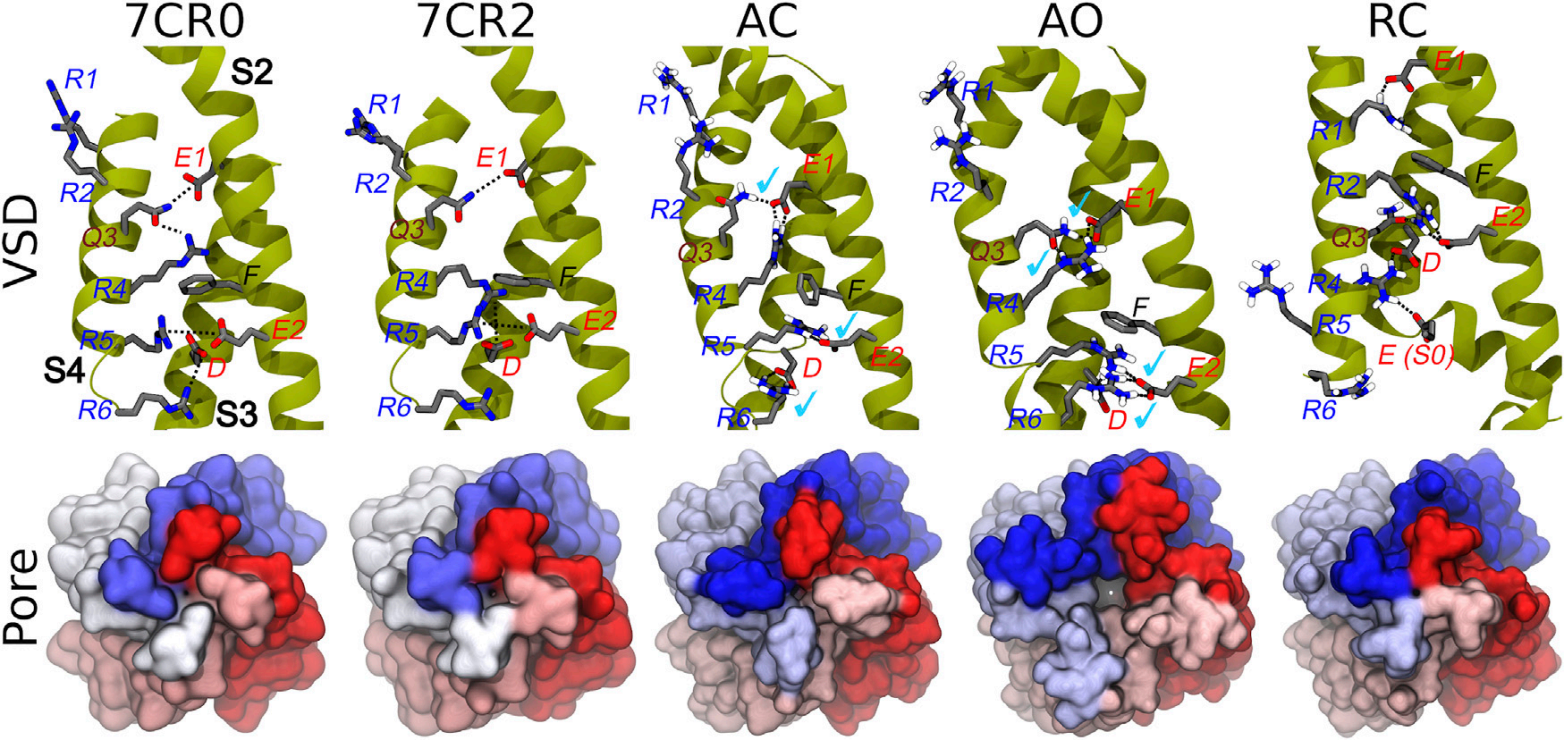
Conformations of the VSD domain. Top row: Key interactions established between charged/polar residues of the S1-S4 helices in the apo and RTG-bound experimental structures, followed by the three models described in this work. Helices S2, S3, and S4 are shown in dark yellow ribbons, while the sidechains of key residues are represented by sticks colored by atom type. H-bonds/salt bridges are indicated by black dotted lines. Light blue check marks identify interactions present in the apo experimental structure (7CR0) and reproduced in the homology models. Bottom row: Comparison among the molecular surfaces (bottom view from the intracellular side; monomers colored differently) of the channel in the experimental and modelled structures generate in this work.

The homology models of the AC, AO, and RC conformational states of the transmembrane region of the tetrameric KCNQ2 channel were generated according to the protocol described in the Methods section. When building the models, the recently published structure of the human KCNQ2 ion channel in the AC state (PDB ID: 7CR0) was unavailable, and the more reliable template was the AC cryo-EM structure of the KCNQ1 ion channel (PDB ID: 5VMS), featuring a high sequence similarity with KCNQ2 (Supplementary Table S1). The AC tetramer resulting from this modelling displays a closed pore and an activated VSD domain: the C_α_-RMSDs values calculated for these domains taking the KCNQ2 structure 7CR0 as reference were 1.0 and 1.6 Å respectively (Supplementary Figure S1), indicating a good agreement between our model and the experimental structure. Moreover, characteristic interactions between E1 on helix S2 with Q3(S4), E2(S2) with R5(S4), and D(S3) with R6(S4), present in the experimental structure, are reproduced in our model (Figure 2). As expected, the access to the pore through the activation gate, lined by residues A306, A309 and S314, is closed.

The AO and RC states were built using as templates the new and refined models of the KCNQ1 channel in the corresponding structures (Kuenze et al., 2019). Also in this case, the resulting models are overall reasonable (Figure 2 and Supplementary Figure S1). In particular, in the AO state the VSD domain reproduces all the key interactions between R and E/D aminoacids sitting on helices S4 and S2, respectively. In contrast, in the resting state the VSD domain features interactions between E1(S2) and R1(S4), E2(S2) and R2(S4), which imply a downward shift of S4 by about 3 helical turns compared to the conformation assumed in the activated state. The pore is clearly closed also in the RC state (Figure 2), while it features a channel leading from the intracellular side to the central cavity (bottleneck radius of 2.46 Å near A309) in the AO state. All models were validated using the MOLPROBITY webserver, with scores of 1.48 (AO), 1.59 (RC), and 1.84 (AC), placing them respectively in the 96th, 93rd, and 84th percentiles (out of 27,675 structures). These values are comparable to that of 1.57 and 1.59 (93rd percentile) obtained respectively for the same region extracted from the cryo-EM structure of KCNQ1 (5VMS) used as structural template and for the cryo-EM structure of KCNQ2 in the AC state (7CR0).

### Characterization of Channel Dynamics in the AC, AO, and RC States

Using the homology models discussed in the previous section as starting structures, we performed 9 independent MD simulations for each state, for a total production trajectory of ∼6 μs in length. In all cases the trajectories were relatively stable, with C_α_-RMSDs from the initial structure stable around 4 Å (Supplementary Figure S2). These values are very similar to that found by Kuenze et al. in their recent computational work on KCNQ1 (Kuenze et al., 2019). Note that, in the absence of soft restraints between K^+^ ions and residues of the Pore-forming region, the number of ions simultaneously present within that region oscillates between 2 and 4. In this work we will not discuss further this aspect, while noting that the removal of the restraints had a limited impact on the overall stability of the protein (Supplementary Figure S2).

Given the recent availability of the experimental structure of the apo form of the channel in the AC state, we compared the structural features of the three simulated systems taking that structure (7CR0, PDB code) as a reference. The distribution of the protein C_α_-RMSDs across the cumulative production trajectories is sharply peaked around 3 Å for the AC model. This confirms that our model is not only stable during μs long MD trajectories, but samples conformations close to the experimental structure resolved for this state (Supplementary Figure S3). As expected, the RC and the AO states sample conformations that are more distant from the AC experimental structure (peaks around 6 and 8 Å, respectively; Supplementary Figure S3, upper panel). Similar distributions plots calculated separately for each VSD domain and for the Pore confirm the overall good reproduction of the structural features at the level of key regions in every conformational state (Supplementary Figure S3, middle and lower panel, respectively). Consistently with these results, the interactions characterizing the active and resting states of the VSD domain, reproduced in the homology models from which the MD simulations begun, are well conserved in at least one domain of all (66%) replicas of the AO/C (RC) states (Table 1 and Supplementary Tables S2–S4). Note that up to three VSD domains assumed simultaneously an active state conformation along the simulations of the AO state (Table 1). To obtain further structural insights into the KCNQ2 channel in the different states, we performed a cluster analysis on the cumulative production trajectory, using as metric the distance-RMSD among the C_α_ atoms of the protein.

**TABLE 1:** Key interactions characterizing the active and resting states of the four VSD domains of KCNQ2 reproduced along the MD simulations of the apo protein. Interactions were considered in place if recorded for at least 50% of the simulated time (production run). For each replica, the number of interactions occurring in each of the four domains is reported, along with the numbers of active or resting domains (defined in this way if the number of key interactions occurring simultaneously amounts to 3 or more).

			Key interactions in active state																					Key interactions in resting state																				
			D-R5				E1-Q3				E1-R4				E2-R5				E2-R6				Interactions per VSD domain (# active domains)	D-R4				E-R4				E1-R1				E2-R2				R2-Q3				Interactions per VSD domain (# resting domains)
System		VSD domain	1	2	3	4	1	2	3	4	1	2	3	4	1	2	3	4	1	2	3	4		1	2	3	4	1	2	3	4	1	2	3	4	1	2	3	4	1	2	3	4	
AO	Replica	1								•				•				•				•	4 (1)																					
		2					•	•			•	•			•	•				•			4, 3 (2)																			•		1
		3						•		•		•		•		•		•		•		•	4, 4 (2)																				•	1
		4						•				•				•				•			4 (1)																					
		5						•			•	•			•	•				•			4, 2 (1)																					
		6						•				•				•				•			4 (1)																					
		7					•				•				•								3 (1)																					
		8										•				•				•			3 (1)																					
		9								•			•	•		•	•	•		•		•	4, 2, 2 (1)																					
RC	Replica	1																																								•		1
		2																												•					•				•			•	•	3, 2 (1)
		3																												•					•							•	•	2, 2
		4																											•	•		•			•	•			•			•		2, 2, 2, 1
		5																											•	•				•	•			•	•			•		4, 2, 1 (1)
		6																												•			•		•		•		•			•	•	3, 2, 2 (1)
		7																												•					•				•			•	•	3, 2 (1)
		8																												•				•				•				•	•	4, 1 (1)
		9																												•					•				•			•	•	3, 2 (1)
AC	Replica	1								•				•			•	•			•		3, 2 (1)																					
		2						•		•		•		•		•		•		•			4, 3 (2)																					
		3						•			•	•			•	•					•		4, 2 (1)																					
		4					•	•		•	•	•		•	•	•		•		•			4, 3, 3 (3)																					
		5					•				•				•								3 (1)																					
		6					•			•	•			•	•			•					3, 3 (2)																					
		7						•				•		•		•		•		•			4, 2 (1)																					
		8						•		•		•		•		•		•					3, 3 (2)																					
		9							•	•	•	•	•	•	•	•	•	•		•	•		4, 4, 2, 2 (2)																					

The results indicate a decreasing structural variability for the AO, AC, and RC states (Supplementary Table S5) and confirmed that each simulation maintains the intended state without evolving to any of the other ones. Indeed, the conformations within each simulation are markedly closer to the initial model of the corresponding state than to the two other ones (Supplementary Figure S4).

Next, we analysed the features of the binding site of the anticonvulsant drug RTG, targeting KCNQ2-5 channels (Kim et al., 2015). Since the structure of RTG bound to the KCNQ2 channel in the AC state (Li et al., 2021) was not published when this study was designed, we identified the putative residues lining this binding site in KCNQ2 based on the information available from literature (see Methods section). Importantly, that region compares very well with the experimental binding site reported by Li et al. with 8 out of 11 residues lining that site being also included in our selection. This essentially expands in this work by a few residues the true binding site for RTG (Figure 1). Moreover, while the binding of RTG has been characterized for the AC state of KCNQ2, no structural information is available regarding the binding of the modulator to the RC and AO states. Therefore, studying the dynamical features of this site on all conformational states of this channel could provide additional insights into the mechanism of action of RTG.

**FIGURE 3:**
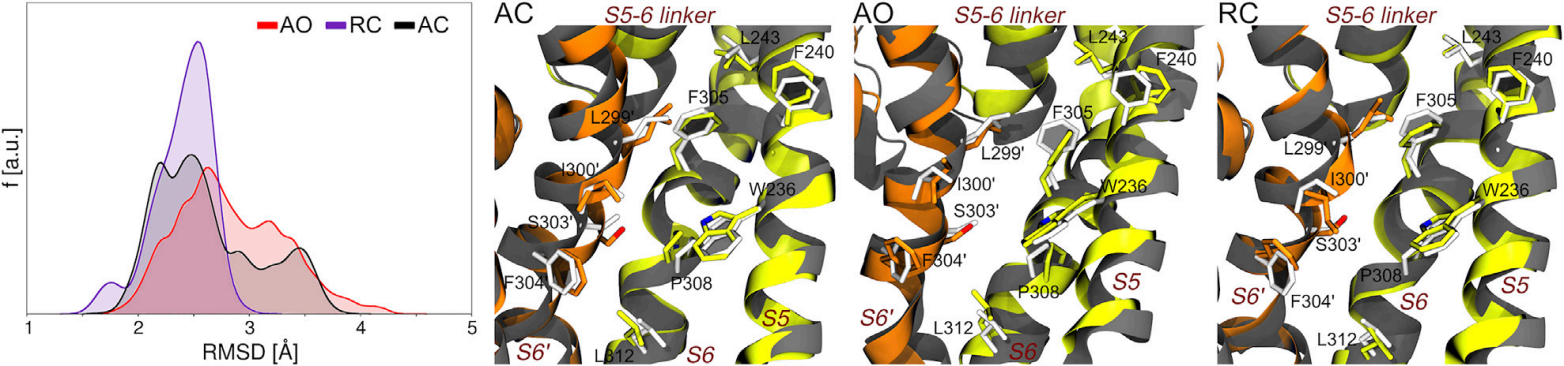
Sampling of bound-like conformations of the RTG binding site (see Figure 1) during the MD simulations of unbound KCNQ2 in the three states considered in this work. The graph on the left displays the RMSD distributions (calculated on all heavy atoms of the residues lining the experimental RTG binding site) extracted from the corresponding MD simulations. The three pictures on the right of this graph show the structures featuring the lowest RMSD from the experimental geometry of the RTG site in each simulation. The S5, S6, and S5-6 linker helices from one monomer are shown as greenish yellow ribbons, while the S6 helix from the adjacent monomer (S6′) is colored in orange. Residues from each monomer are shown as sticks colored by atom type (carbon atoms colored as the ribbons). The experimental conformation is shown in grey color.

Along this line, we first assessed if and to what extent the precise geometry of the binding site hosting RTG in the experimental structure is reproduced during the MD simulations of the AC, AO, and RC conformational states of KCNQ2. As can be seen in Figure 3, bound-like conformations are sampled along all simulations with the RMSD calculated at the RTG site reaching values as low as 1.3, 1.5, and 1.7 Å for the RC, AC, and AO conformational states, respectively. A significant fraction of conformations with RMSD values lower than 2 Å were sampled in the RC and, to a slightly lower extent, AC states.

However, it is worth noticing that also the AO state, starting from a conformation distant 3.6 Å from the experimental geometry (2.3 and 2.4 Å for RC and AC, respectively), also assumed a non-negligible fractions of bound-like conformations.

To further assess the possibility of drug binding at this site in all KCNQ2 conformational states, we estimated the fraction of druggable geometries sampled along the trajectories of each of them. Namely, we calculated the druggability score D on a set of structures obtained from an additional cluster analysis performed on the putative RTG binding site identified in Figure 1 (see Supplementary Table S5). Such analysis was justified not only because of the larger conformational variability of the RTG site in the AO state (Figure 3), but also in view of: 1) previous findings by Kim and co-workers (Kim et al., 2015), reporting two possible orientations assumed by the residue W265 of the KCNQ3 channel—equivalent to W236 in KCNQ2—when in complex with RTG; 2) the change in the network of residues involved in interactions with RTG, which was suggested to occur upon switch of the channel from a closed to an open state (Syeda et al., 2016). According to the software *fpocket* ((Le Guilloux et al., 2009), see Methods section), a site is defined as druggable (that is, likely to accommodate a drug-like molecule) if D is larger than 0.5. The results of this analysis (Table 2) show that: 1) for each state there are at least two druggable RTG binding sites; 2) in the RC state, three out of four RTG binding sites sample druggable conformations, and their relative population is the highest among the three states; 3) the AO state features the lowest relative population of druggable conformations, although their scores are on average the highest among the series. The occurrence of druggable conformations of the RTG binding sites in all states of the channel could have implications for the action mechanism of RTG and other modulators.

**TABLE 2:** Druggability index D calculated with fpocket on each of the four binding sites of RTG in the three different states of the KCNQ2 ion channel. For each site, the average value (weighted by the relative cluster population) of D and its standard deviation are reported, together with the normalized frequency f_N_ of conformations identified as druggable (calculated as sum of normalized fractions of cluster population).

	Channel state					
	AO		RC		AC	
Site	D	f_N_	D	f_N_	D	f_N_
1	0.97 (0.03)	0.23	0.70 (0.19)	0.59	-	-
2	-	-	-	-	-	-
3	0.92 (0.10)	0.17	0.66 (0.02)	0.40	0.71 (0.17)	0.40
4	-	-	0.83 (0.03)	0.44	0.92 (0.05)	0.40

### Characterization of RTG Binding Onto Putative Binding Site

To identify possible binding modes of RTG on KCNQ2 in the AO and RC conformational states, we performed molecular docking calculations on different conformations of the four putative binding sites located on the Pore domain of the channel. Namely, ensemble docking was performed on 42 and 16 structures in the AO and RC states, respectively (see Supplementary Table S5 and the Methods section). At least one pose resembling the experimental binding mode was reproduced for both states, although the orientation of the residues lining the experimental binding site was virtually reproduced only in the AO state (Supplementary Figures S5, S6). Note that the docking score appears to be relatively insensitive to the conformation assumed by the channel, which points to the possibility of stable RTG binding to the protein bearing an open Pore. To further assess this possibility, starting from the top eight non-overlapping docking poses within each site (selected by visual inspection), we performed 24 1 μs-long simulations for the AO-RTG and for the RC-RTG complexes (three replicas for each pose, for a cumulative time of 48 μs). Given the unavailability of an experimental pose of RTG at the time of running our simulations, we relied on usual criteria (high docking score and pose occurrence) to select plausible conformations of the complexes.

Among the poses selected as starting structures for MD simulations, the ones resembling more closely the experimental structure of RTG featured RMSD values of 4.7 Å and 3.5 Å for the RC and AO states, respectively (Supplementary Figures S5, S6). Out of the 24 MD simulations performed for each of the AO and RC states, respectively 12 and 20 resulted in stable complexes between RTG and the channel, confirming the possibility of complex formation in different KCNQ2 states. Remarkably, in a few (one) simulations of the AO(RC)-RTG complex, the RTG molecule evolved towards a binding conformation closely resembling the experimental one (minimum RMSD values amounting to 0.8 and 0.7 Å from starting values of 4.4 and 4.8 Å, respectively; see Supplementary Figures S7, S8).

More importantly, the most populated cluster of the RTG-AO complex is the one featuring a RTG binding mode essentially equivalent to the experimental one, associated with a relatively high affinity compared to most clusters (Table 3). This nicely validates our overall modelling and simulation strategy, and points to the possibility for this modulator to interact with states other than AC without the need to significantly alter its contact network.

**TABLE 3:** Pseudo binding free energies and structural deviations from the experimental pose calculated for the top 10 clusters of the complexes between RTG and KCNQ2 in AO and RC conformational states. Standard deviations are reported in parentheses.

State Clustermovetoleft	RTG-AO		RTG-RC	
	ΔG [kcal/mol]	$RMSD_{7CR2}^{RTG}$ [Å]	ΔG [kcal/mol]	$RMSD_{7CR2}^{RTG}$ [Å]
0	−30.8 (3.9)	1.5 (0.4)	−28.6 (3.1)	4.6 (0.3)
1	−22.2 (2.9)	4.5 (0.4)	−22.0 (3.8)	5.3 (0.4)
2	−26.4 (2.5)	4.8 (0.3)	−26.2 (2.4)	9.2 (0.1)
3	−36.2 (3.2)	9.0 (0.2)	−25.3 (3.9)	9.7 (0.6)
4	−33.7 (3.5)	4.7 (0.5)	−21.5 (3.4)	3.5 (0.3)
5	−28.1 (2.6)	11.4 (0.5)	−16.0 (2.2)	9.6 (0.5)
6	−25.9 (2.5)	4.5 (0.4)	−24.3 (3.5)	5.9 (0.4)
7	−22.1 (2.9)	7.1 (0.3)	−22.5 (3.3)	5.0 (0.5)
8	−30.7 (2.6)	4.7 (0.3)	−26.5 (3.8)	7.8 (0.3)
9	-−29.2 (2.3)	9.1 (0.3)	−22.1 (2.7)	5.6 (0.3)

To assess more quantitatively the sampling of conformations of RTG resembling the experimental geometry in the RTG-AC complex, we performed a cluster analysis on the cumulative trajectory of each simulated system. In addition, we estimated the stability of the binding poses corresponding to the 10 most populated clusters via MM/GBSA calculations (see Methods section). We notice that, on average, the affinity of the ligand is higher towards the channel in the AO state than the RC one (although the differences are minor and should be taken as a qualitative indication about binding propensity of RTG). While experimental binding geometries were recovered also in the simulations of the RTG-RC complexes (Supplementary Figure S8), they were not picked up in the top 10 conformational clusters (the lowest RMSD being 3.5 Å for the 5th cluster representative, see Table 3).

**FIGURE 4:**
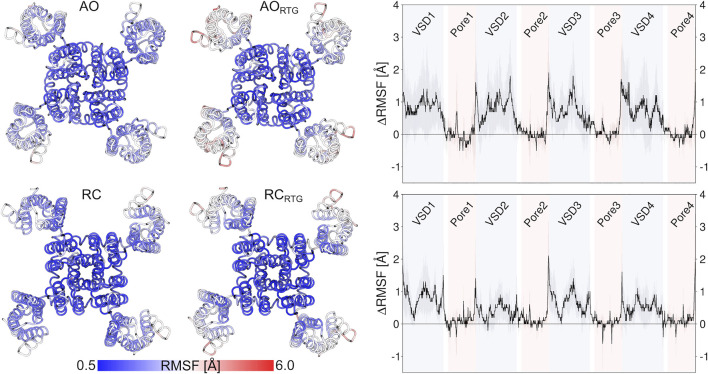
Effect of RTG binding on the flexibility of KCNQ2, measured in terms of average RMSF calculated on the stable MD simulations discussed in the main text after alignment of the Pore domain. The pictures on the left illustrate the increase in the RMSF values of the VSD domains after binding of RTG to the AO (upper panel) and RC (lower panel) states. The graphs on the right site help to better quantify such an increase.

As we don’t know if the binding mode of RTG is fully conserved across the different states of the channel, in the following we discuss our results in terms of average structural and dynamical, properties across the stable trajectories of the complexes. This allows evaluating our findings on the effect of RTG binding on KCNQ2 free of any experimental bias. First, we found that binding of RTG alters the dynamics of the channel; namely, it slightly rigidifies the Pore domain, and moreover, it increases the flexibility of the VSD domains with respect to the unbound protein, particularly in the AO state (Figure 4). This result is in agreement with the findings reported in (Li et al., 2021), showing an increase in the values of the B-factor off the VSD domains upon binding of four RTG molecules. Our data suggest that such an increase represents a general consequence of RTG binding to any conformational state of the channel. Next, to assess if the enhanced flexibility of the VSD domains resulting from RTG binding affects their propensity towards active conformations, we analysed the occurrence of specific interactions characterizing VSD active and resting (Table 4, Supplementary Tables S6, S7). Interestingly, binding of RTG to the RC state induces a decrease from 66 to 50% in the percentage of MD simulations featuring at least one VSD domain in the resting state (although no active VSD domain was detected, possibly due to the longer timescales of this event compared to the length of our simulations, and to the presence of only one compound instead of four as in the cryo-EM structures). In contrast, binding of the modulator to the AO state does not significantly alters the relative occurrence of activated vs. resting conformations, consistently with the proposed mechanism of action (Gunthorpe et al., 2012; Li et al., 2021).

**TABLE 4:** Key interactions characterizing the active and resting states of the four VSD domains of the KCNQ2 channel reproduced during the MD simulations of the RTG-channel complexes. See Table 1 for details.

			Key interactions in active state																					Key interactions in resting state																				
			D-R5				E1-Q3				E1-R4				E2-R5				E2-R6				Interactions per VSD domain (# activated domains)	D-R4				E-R4				E1-R1				E2-R2				R2-Q3				Interactions per VSD domain (# resting domains)
System		VSD domain	1	2	3	4	1	2	3	4	1	2	3	4	1	2	3	4	1	2	3	4		1	2	3	4	1	2	3	4	1	2	3	4	1	2	3	4	1	2	3	4	
RTG-AO	Replica	1							•	•			•	•			•						3, 2 (1)																			•		1
		2						•		•		•		•				•					3, 2 (1)																			•	•	1, 1
		3								•				•				•					3 (1)																			•	•	1, 1
		4						•		•		•		•		•		•		•			4, 3 (2)																			•		1
		5								•		•		•	•			•					3, 2 (1)																			•	•	1, 1
		6					•	•			•	•		•	•	•		•		•			4, 3, 2 (2)																			•		1
		7							•			•	•							•	•		3, 2 (1)																			•		1
		8						•				•				•				•			4 (1)																					
		9					•		•	•	•		•	•	•		•	•					3, 3, 3 (3)																			•	•	1, 1
		10						•				•				•				•			4 (1)																			•	•	1, 1
		11					•	•	•	•	•	•	•	•		•	•	•			•	•	4, 4, 3, 2 (3)																			•	•	1, 1
		12						•		•		•	•	•		•	•				•	•	3, 3, 3 (3)																				•	1
RTG-RC	Replica	1																											•	•												•	•	2, 1, 1
		2																											•	•				•			•	•		•		•		4, 2, 1 (1)
		3																																								•	•	1, 1
		4																												•		•				•						•		2, 2
		5																												•		•	•			•	•			•			•	3, 2, 1, 1 (1)
		6																											•			•				•								2, 1
		7																																						•				1
		8																												•	•				•					•			•	3, 1, 1 (1)
		9																												•	•	•		•		•		•			•		•	3, 2, 2, 1 (1)
		10																										•	•	•	•	•	•			•	•				•	•	•	4, 3, 2, 2 (2)
		11																										•	•	•			•			•	•	•		•				3, 3, 2 (2)
		12																												•										•	•			2, 1, 1
		13																												•		•			•	•			•	•			•	3, 3,1 (2)
		14																												•		•	•			•	•					•	•	2, 2, 2, 1
		15																												•			•		•		•		•					2, 2, 1
		16																											•			•	•			•	•							3, 2 (1)
		17																											•	•				•		•		•				•	•	4, 1, 1, 1 (1)
		18																											•	•						•							•	1, 1, 1, 1
		19																											•	•		•	•	•		•	•	•		•		•		4, 3, 3 (3)
		20																											•	•		•										•	•	1, 1, 1, 1

**FIGURE 5:**
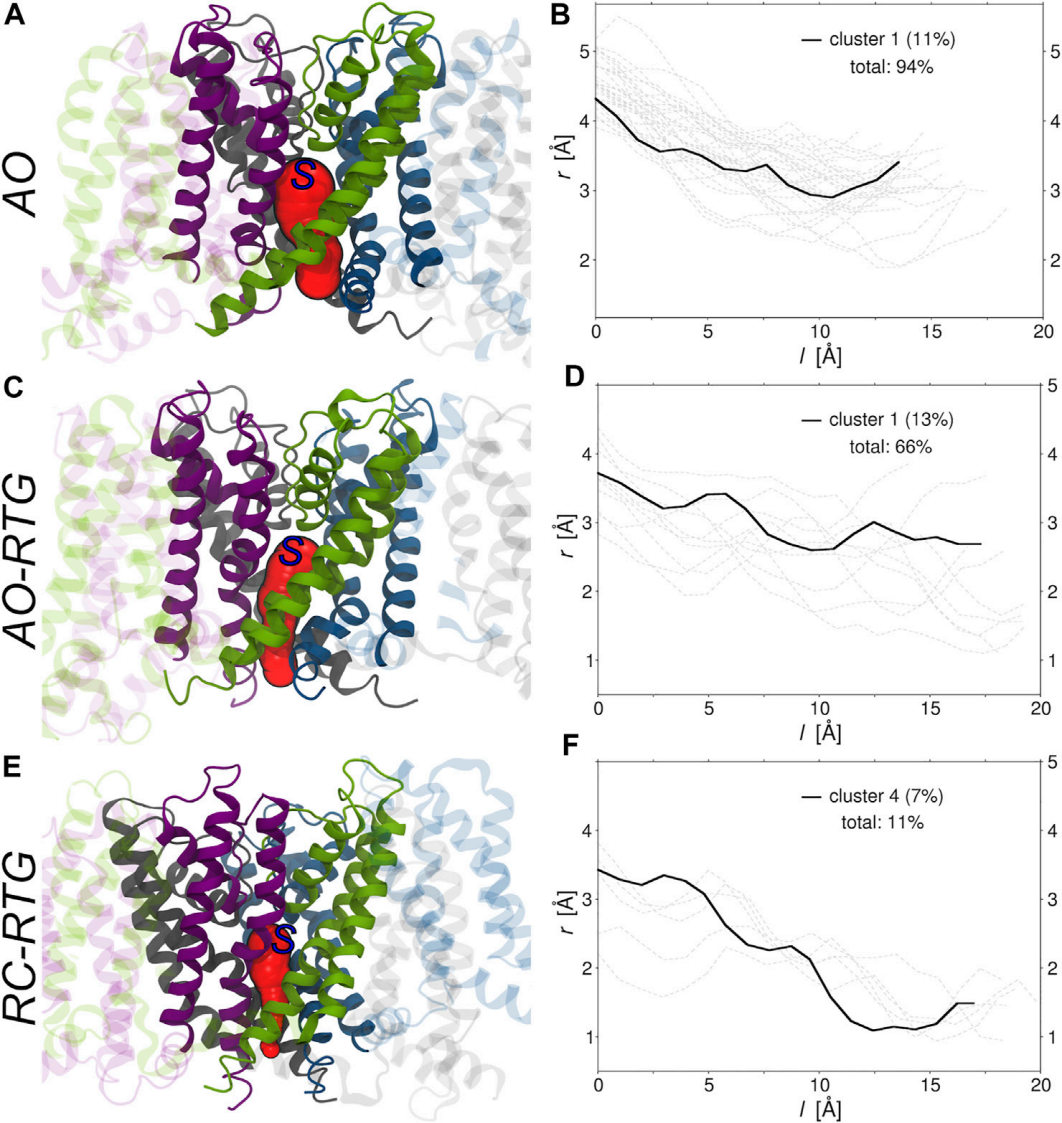
Morphologies of the tunnels leading from the center to the cytoplasmic end of KCNQ2. **(A,C,E)** Tunnels (red surfaces) found in the main (A, C) and fourth (E) conformational clusters extracted from the cumulative trajectories of the channel in the AO state, AO bound to RTG, and RC bound to RTG, respectively. Each monomer is shown in ribbons colored differently, with the Pore domain solid and the rest of the protein transparent. The starting point set for tunnel detection is approximately indicated by a blue capital S letter. **(B,D,F)** Radii (r) vs. path length (l). The profile of the tunnel found in the most populated non-closed cluster (population in parenthesis) is shown by a black solid line, while the profiles associated to the tunnels found in the remaining clusters are shown by gray dashed lines. The total percentage of clusters bearing a tunnel is also reported in each graph.

Finally, we investigated if and to what extent the binding of RTG alters the morphology of tunnels leading from the center of KCNQ2 (beneath the selectivity filter) to its cytoplasmic gate. To this end, we run the software CAVER (Chovancova et al., 2012) on the representative structures of all the conformational clusters extracted from the cumulative trajectory of each simulated system (Supplementary Table S5). Consistently with experiments, in the absence of RTG virtually all conformations of the protein bear a tunnel in the AO state; in contrast, only closed conformations were found for the AC and RC states. Tunnel bottlenecks were found near the cytoplasmic end of the protein, with radii ranging from 1.9 to 3.5 Å (2.9 Å for the representative of the most populated cluster; see Figure 5). Binding of RTG to the channel in the AO state did not induce large structural changes in the morphology of the Pore, with 2/3 of the conformations still bearing a tunnel. More relevantly, binding of just one RTG molecule to the RC state seems to alter the conformational equilibrium of KCNQ2 in favour of conformations bearing an open Pore. Indeed, about 1/10 of the cluster representatives feature a tunnel with a bottleneck radius ranging between 1 and 1.2 Å.

While the limitations of our approach do not allow to assess the long-term effects of RTG binding on KCNQ2, the early steps of channel opening induced by ligand binding caught by our analysis shed light on the mechanism of action by this compound. Overall, our findings support an allosteric mechanism in which binding of RTG to the channel would promote its transition towards the AO conformational state. Such conformational transition could occur, according to our data, without the need for drastic rearrangements of RTG within the binding site recently validated by Li and co-workers (Li et al., 2021).

## Concluding Remarks

KCNQ2 is a main molecular determinant of the M-current, a widespread K^+^ current regulating neuronal excitability. Because of their fundamental role in regulating cellular excitability, this channel in its heterotetrameric form with KCNQ3 is implicated in several human disease conditions, including epilepsy, pain, migraine, arrhythmias, sensory dysfunction, and metabolic illnesses. The KCNQ2/KCNQ3 opener retigabine represents an attractive compound for the treatment of these diseases. Structural information about the molecular interactions of this compound with KCNQ2 along the conformational cycle (the various states) of the channel can be highly informative to drug design efforts.

However, apart the recent cryo-EM structure of AC KCNQ2, alone and in complex with RTG (unavailable when this work was undertaken), to date there are no KCNQ2 structures available in the RC and AO states. For these reasons our efforts were aimed at generating models of all three states and characterise their dynamics and interaction with RTG. Conformations extracted from the molecular dynamics simulations were used to dock RTG into the AO and RC states and compared with the experimental structure of the KCNQ2 in the AC state complexed with RTG. All the generated models of both the apo structures in various states as well as the complexes with RTG were subjected to MD simulations, for a total simulated time of ∼50 μs. The analysis of the trajectories confirmed the stability of the apo structures as well as the AO and RC structures complexed with RTG. Our results on the effect of binding of one RTG molecule to the AO and RC states are in line with experimental findings on the channel in the AC state bound to four drug molecules. Overall, our data suggest that RTG can bind to the same site of the channel, and maintaining the same orientation, independently of the KCNQ2 (and thus Pore domain) conformation/state. Furthermore, our simulations of the RTG-bound states unveil a tendency of the channel towards increased flexibility of the VSD domains and sampling of conformations corresponding to an open Pore, which are also in line with experiments. The excellent agreement of our simulations with the experimental AC state structure gives trust into our modelling approach and confidence that the new models of the AO and RC states of this channel are reliable, allowing to uncover new insightful details on the interaction of this channel with RTG.

## Data Availability

The original contributions presented in the study are included in the article/Supplementary Material, further inquiries can be directed to the corresponding authors.
