# Supplementary material for: Molecular Insights Into Binding and Activation of the Human KCNQ2 Channel by Retigabine
Source: Front Mol Biosci. 2022 Mar 3;9:839249. doi: 10.3389/fmolb.2022.839249 (PMC8927717; doi:10.3389/fmolb.2022.839249)
Supplement: Supplementary file 1 [file DataSheet1.PDF]

Supplementary Figures

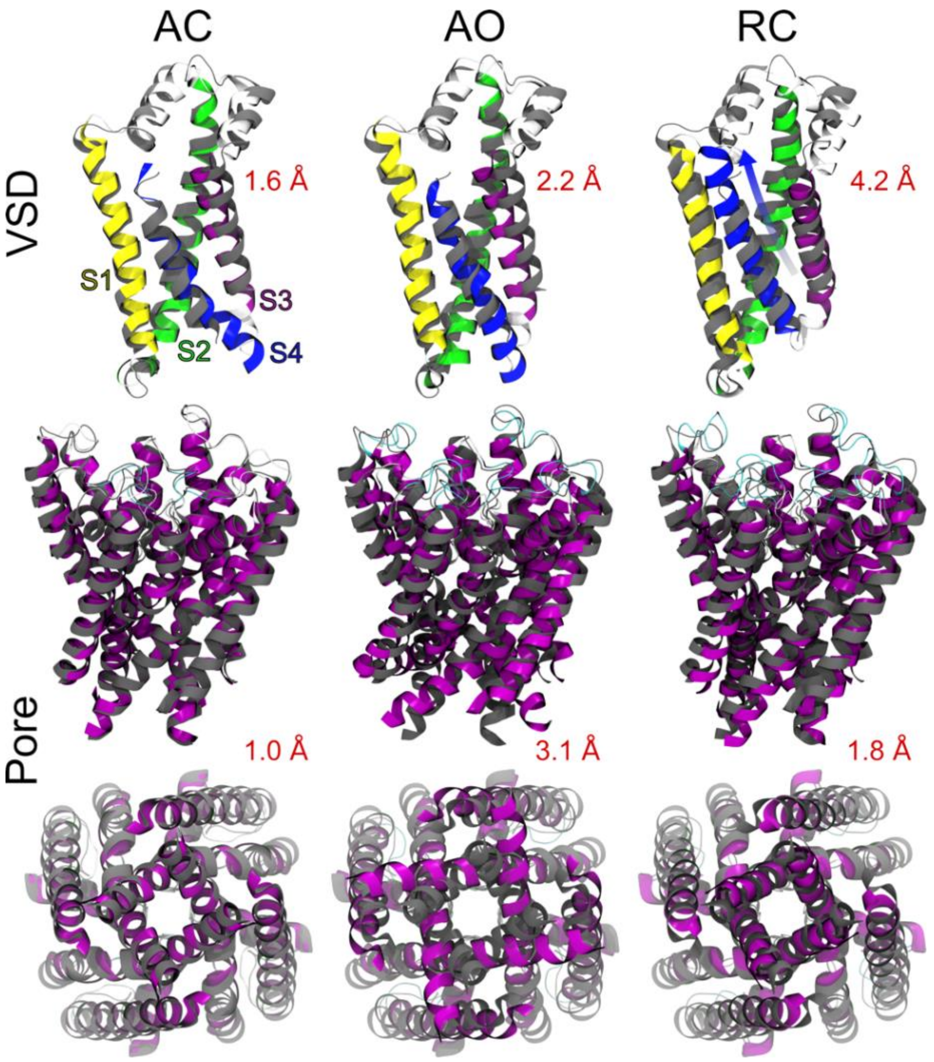

Figure S1. Overall comparison of the structural features of the AC, AO, and RC states modelled by homology. Top row: superposition between the VSD domains (the models are virtually symmetric, thus only one VSD domain is shown) of the models (shiny colored ribbons) and the reference structure 7CR0 (gray ribbons). Helices S1 to S4 are colored differently and indicated by labels in the upper left picture. The sliding of S4 in the RC state is highlighted by an arrow on the right side of the helix). C_α-RMSD values between the VSD domains of the model and of the experimental structure are reported on the right of each image. Middle and bottom rows: Side and bottom (gate facing the reader) views of the superposition between the pore domains of the homology models (ribbons colored according to the secondary structure) and the reference experimental structure (gray ribbons). Also in this case, C_α-RMSD values from the experimental structure are reported on the right of each image.

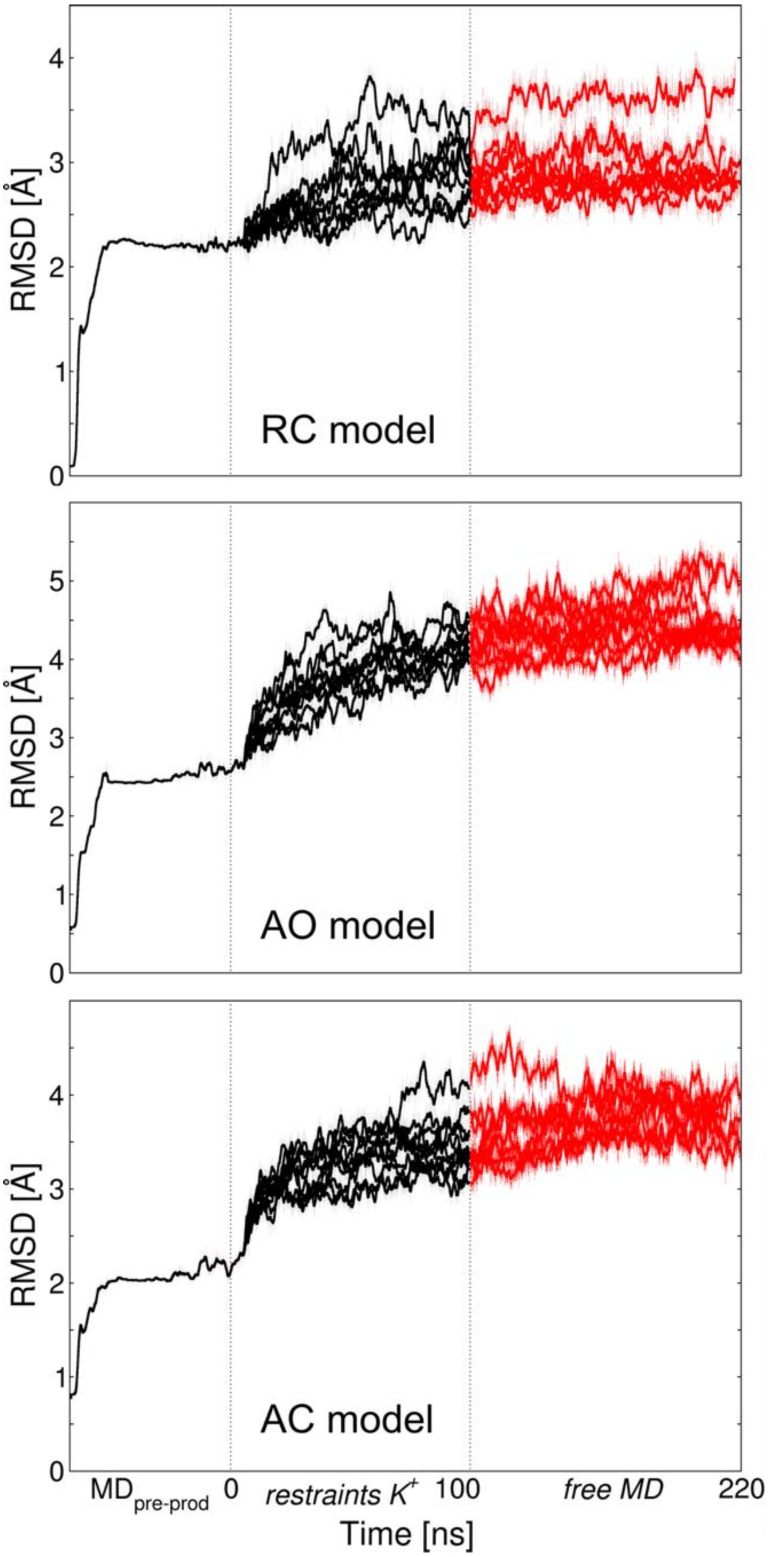

Figure S2. Cα-RMSD profile of the protein channel calculated from 9 independent trajectories for each of the three RC, AO, and AC states, taking the corresponding homology models as reference structures.

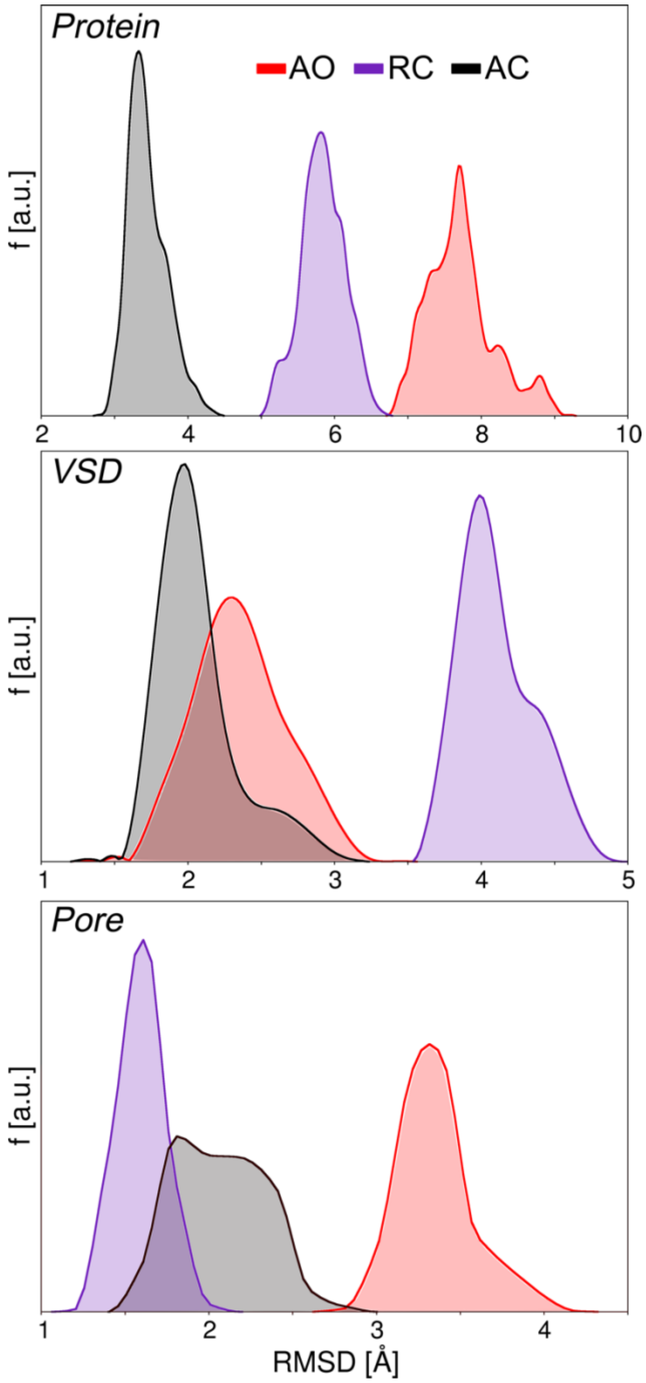

Figure S3. C_α-RMSD distributions of the whole protein, the VSD, and the Pore domain, calculated on the cumulative trajectories of the apo channel in the AO, RC, and AC conformational states taking the structure with PDB ID 7CR0 (Li et al. 2021) as reference.

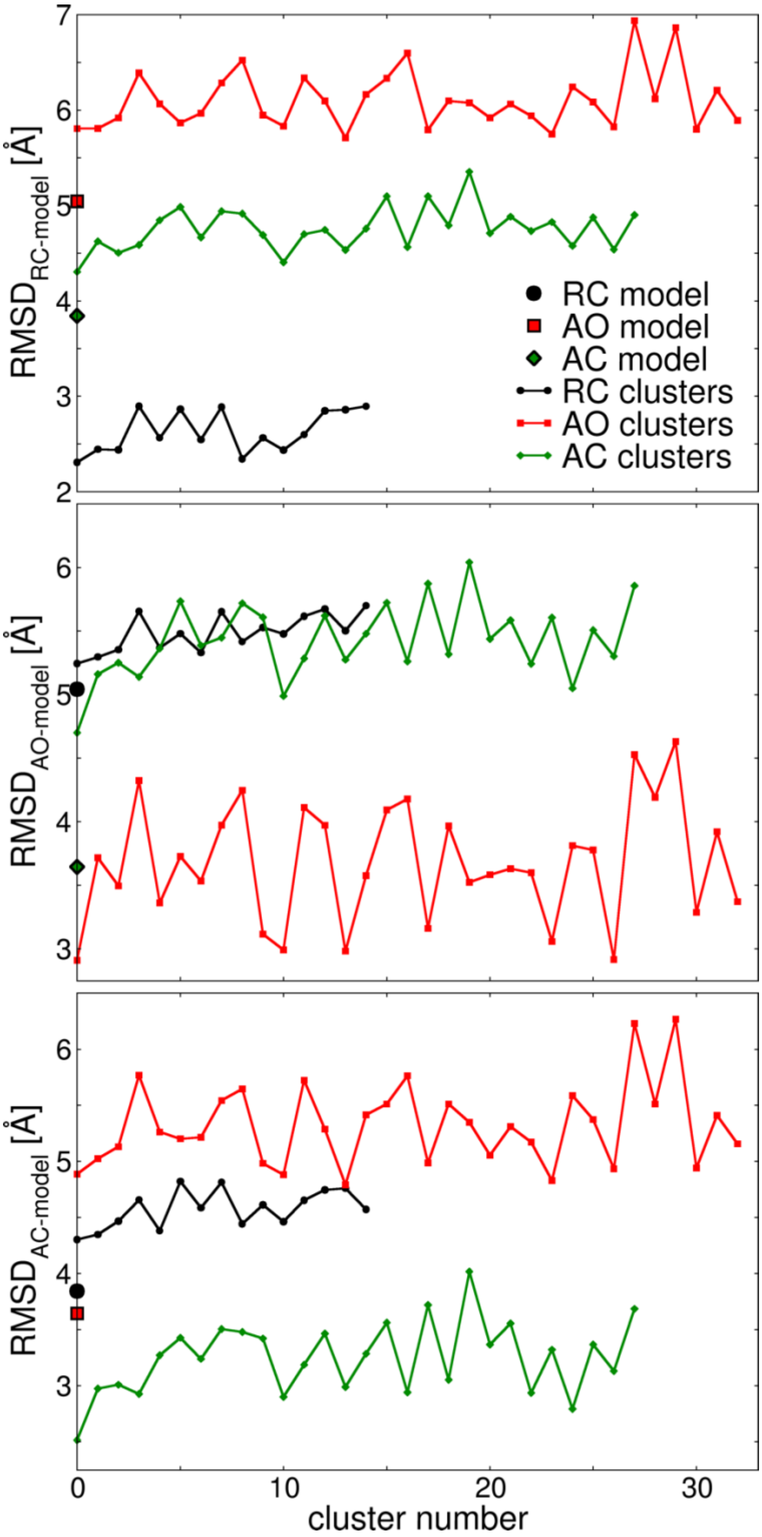

Figure S4. C_{α}-RMSD of cluster representatives extracted from MD simulations of all states with respect to the initial structure used for the simulations of RC (upper panel), AO (middle panel) and AC (lower panel) states.

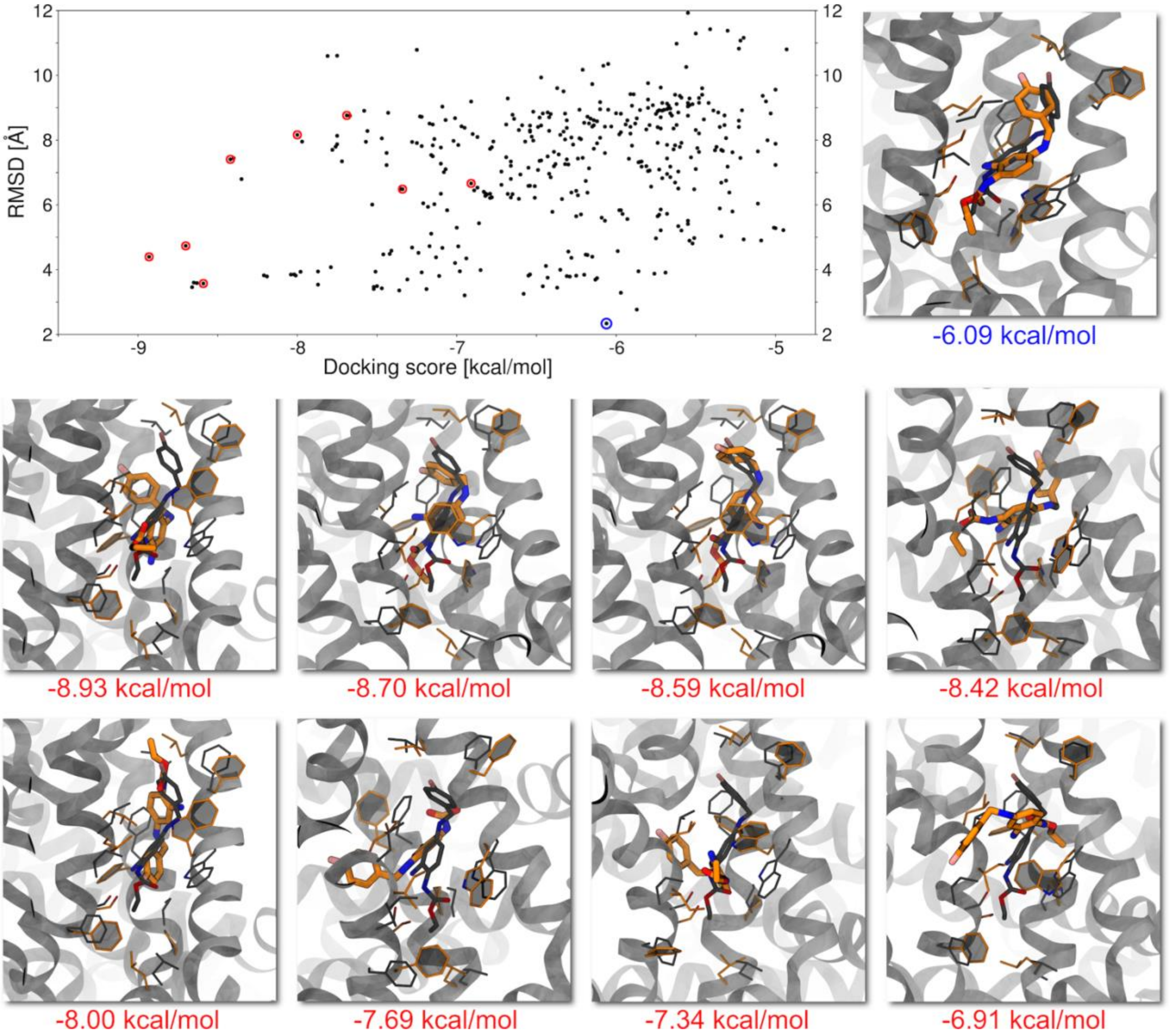

Figure S5. Results of ensemble docking calculations performed on a set of cluster representative structures of the AO conformational state of KCNQ2. The graph on the upper left side of the figure shows the scatter plot of the score vs. RMSD of RTG from the experimental pose (PDB ID: 7CR2). The blue circle identifies the pose featuring the lowest RMSD value; the red circles identify poses selected for refinement through MD simulations. The pose closest to the experimental one is shown in the upper right side of the picture. The protein is shown in grey ribbons; the docking geometry of RTG and binding site residues is shown with sticks colored by atom type (C, N, O in orange, blue, red, respectively), while the experimental conformation is shown by thinner sticks with C atoms colored grey.

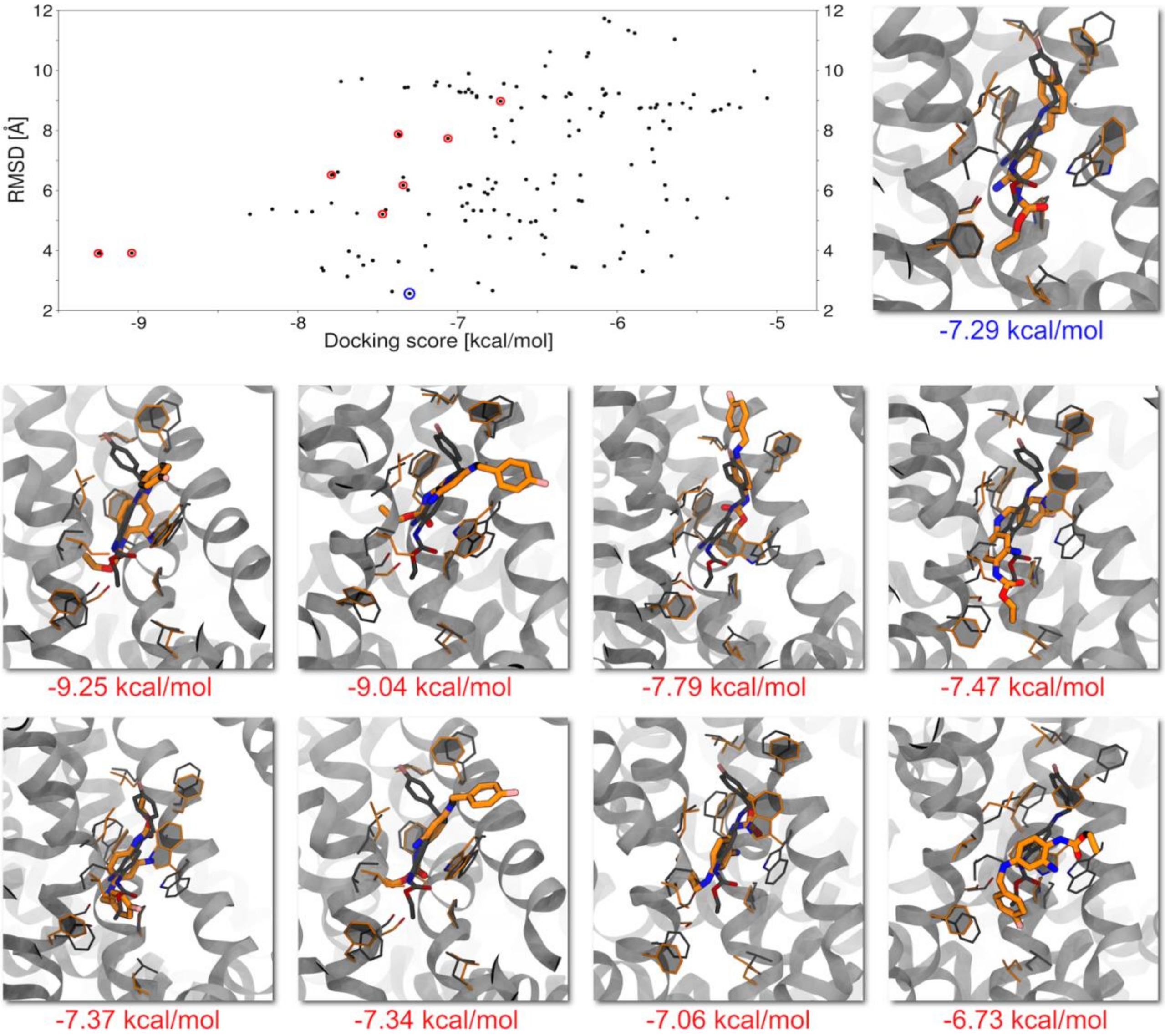

Figure S6. Results of ensemble docking calculations performed on a set of cluster representative structures of the RC conformational state of KCNQ2. The graph on the upper left side of the figure shows the scatter plot of the score vs. RMSD of RTG from the experimental pose (PDB ID: 7CR2). The blue circle identifies the pose featuring the lowest RMSD value; the red circles identify poses selected for refinement through MD simulations. The pose closest to the experimental one is shown in the upper right side of the picture. The protein is shown in grey ribbons; the docking geometry of RTG and binding site residues is shown with sticks colored by atom type (C, N, O in orange, blue, red, respectively), while the experimental conformation is shown by thinner sticks with C atoms colored grey.

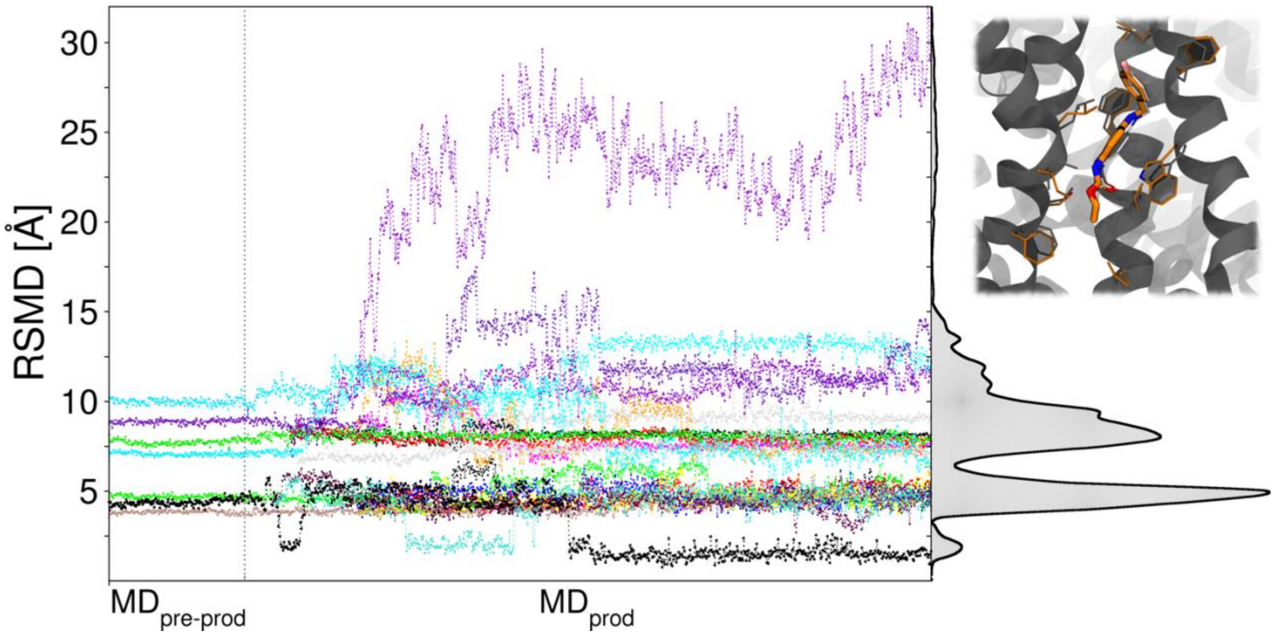

Figure S7. RMSD profiles of RTG along the MD simulations of its docking complexes with KCNQ2 in the AO conformational state, calculated with respect to the experimental pose in 7CR2 after alignment of the binding site. The cumulative distribution is reported on the right side of the graph. The inset image shows the RTG conformation most closely resembling the experimental pose in the AC state.

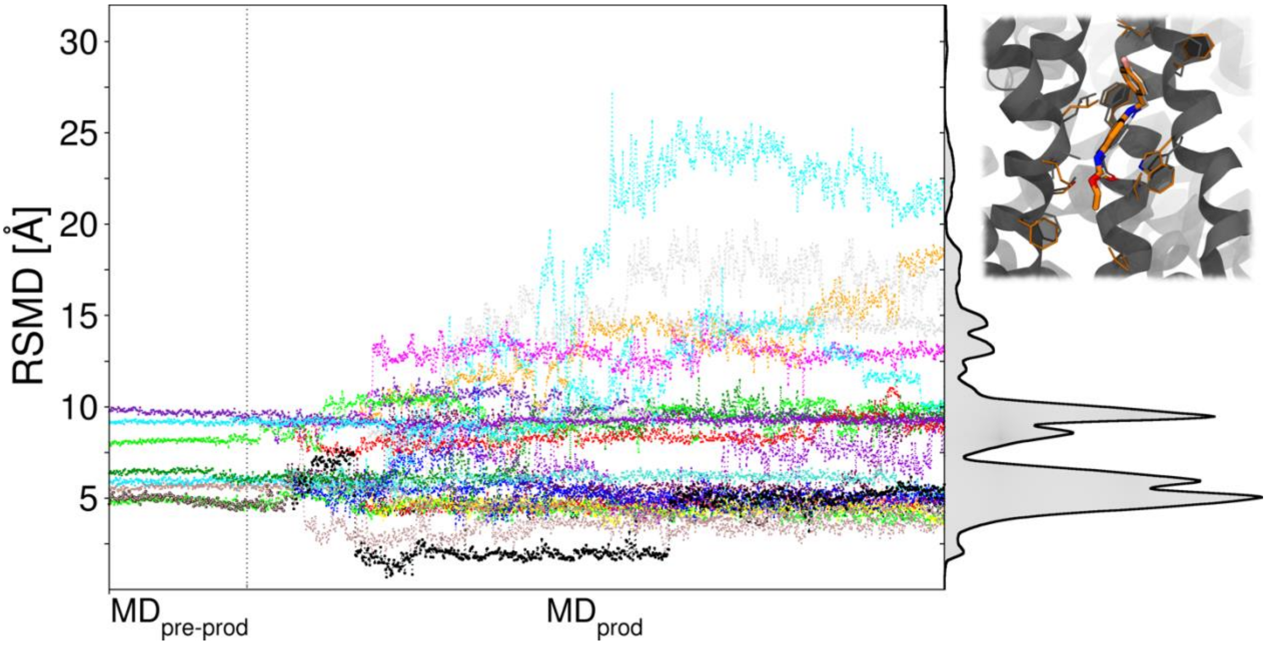

Figure S8. RMSD profiles of RTG along the MD simulations of its docking complexes with KCNQ2 in the RC conformational state, calculated with respect to the experimental pose in 7CR2 after alignment of the binding site. The cumulative distribution is reported on the right side of the graph. The inset image shows the RTG conformation most closely resembling the experimental pose in the AC state.

Supplementary Tables

Template (PDB ID, description)	#AA template (TM region)[1]	Seq. identity[2]	Seq. similarity[2]	Gaps	Notes
KCNQ1 (5VMS)	94-352 (259)	150/259 (57.9%)	193/259 (74.5%)	10/259 (3.9%)	-

1. According to topology definition by UNIPROT and revised through visual inspection and literature; 2. After sequence alignment with Clustal Omega 1.2.4

Table S1. Details of the comparison between the sequence of the S0-S6 stretch in KCNQ2 and KCNQ1.

AC		REP1				REP2				REP3				REP4				REP5				REP6				REP7				REP8				REP9			
VSD domain		1	2	3	4	1	2	3	4	1	2	3	4	1	2	3	4	1	2	3	4	1	2	3	4	1	2	3	4	1	2	3	4	1	2	3	4
L81	E86	0.76	-	-	-	0.83	-	-	-	0.61	-	-	-	0.62	-	-	-	0.75	-	-	0.58	0.23	-	-	0.67	0.69	-	-	0.14	0.26	-	0.71	0.18	0.45	-	-	0.66
Y82	E86	-	0.92	0.93	0.54	-	0.92	0.92	0.83	-	0.90	0.96	0.79	-	0.85	0.59	0.59	-	0.91	0.94	0.13	0.39	0.98	0.62	-	-	1.00	0.86	0.30	0.39	0.82	-	0.36	-	0.89	0.80	0.27
E86	R144	0.99	-	-	-	0.41	-	-	-	0.99	-	-	-	1.00	-	-	-	0.99	-	-	-	0.98	-	-	-	0.99	-	-	-	0.95	-	0.59	-	0.99	-	-	-
E86	R213	0.20	-	-	-	-	-	-	-	0.78	-	-	-	0.99	-	-	-	0.99	-	-	-	1.00	-	-	-	0.99	-	-	-	0.75	-	0.11	-	0.89	-	-	-
C106	Q204	-	0.25	-	-	-	0.14	-	-	-	0.30	-	-	-	0.37	-	-	-	0.54	-	-	-	0.19	-	-	0.18	0.12	-	-	-	0.24	-	-	-	0.19	-	-
S110	R201	0.38	-	1.00	-	-	-	-	0.93	0.25	1.00	-	-	1.00	1.00	1.00	1.00	-	1.00	-	1.00	0.45	0.56	-	1.00	0.66	1.00	-	1.00	1.00	1.00	-	1.00	0.92	0.93	-	1.00
S110	Q204	0.19	0.82	0.97	-	-	0.88	0.70	-	0.64	0.75	0.98	-	-	0.68	1.00	0.70	-	0.47	1.00	0.73	0.16	0.73	0.93	-	-	-	1.00	0.70	0.37	0.81	0.96	0.61	-	-	1.00	-
S113	R201	-	-	-	-	0.54	-	-	-	0.28	-	0.52	-	-	-	-	-	-	-	0.12	-	0.26	0.12	0.18	-	-	-	0.13	-	0.11	-	-	-	0.31	0.35	-	-
E130	T133	-	-	-	-	-	0.58	-	-	-	0.24	-	-	0.15	0.74	-	-	-	-	-	-	-	-	-	-	-	0.54	-	-	-	-	-	-	-	0.26	-	-
E130	I134	-	-	-	0.96	-	0.78	-	0.97	0.94	0.89	-	-	0.90	0.87	-	0.96	0.84	-	-	-	0.95	-	-	0.97	-	0.63	-	0.98	-	0.88	-	0.96	0.95	0.33	0.71	0.99
E130	S179	-	-	-	0.98	-	-	-	0.48	0.82	-	-	-	0.81	-	-	0.29	0.31	-	-	-	0.24	-	-	0.90	-	-	-	0.61	-	-	-	0.29	0.94	-	0.14	0.99
**E130**	**Q204**	-	-	-	0.77	-	0.96	-	0.60	0.15	0.97	-	-	0.86	0.88	-	0.93	0.88	-	-	-	0.97	-	-	0.96	-	0.88	-	0.43	-	0.99	-	0.82	-	1.00	1.00	0.32
**E130**	**R207**	-	-	-	1.00	-	1.00	-	1.00	1.00	1.00	-	-	1.00	1.00	-	1.00	1.00	-	-	-	1.00	-	-	1.00	-	1.00	-	1.00	-	1.00	-	1.00	1.00	1.00	1.00	1.00
T133	F137	-	-	-	0.95	-	0.96	-	0.97	0.92	0.97	-	-	0.91	0.82	-	0.98	0.73	-	-	-	0.95	-	-	0.96	-	0.62	-	0.99	-	0.94	-	0.95	0.94	0.24	0.63	0.99
T133	R207	-	-	0.86	-	-	0.18	-	-	-	0.11	-	-	-	0.26	-	-	0.21	-	-	-	-	-	-	-	-	0.52	-	-	-	-	-	-	-	0.65	0.27	-
V136	E140	-	-	-	0.92	-	0.77	-	0.93	0.92	0.66	-	-	0.96	0.56	-	0.97	0.91	-	-	-	0.99	-	-	0.94	-	0.78	-	0.97	-	0.77	-	0.95	0.83	0.77	0.20	0.81
F137	Y141	-	-	0.70	0.79	-	0.59	-	0.71	0.60	0.52	-	-	0.69	0.35	-	0.71	0.60	-	-	-	0.52	-	-	0.82	-	0.88	-	0.80	-	0.60	-	0.78	0.77	0.77	0.50	0.85
F137	R210	-	-	-	0.14	-	-	-	0.24	0.18	-	-	-	0.12	-	-	0.14	0.15	-	-	-	0.56	-	-	-	-	-	-	-	-	-	-	0.19	0.12	-	-	-
E140	Y95	-	-	-	0.45	-	-	-	0.93	0.86	0.19	-	-	0.98	-	-	0.18	0.97	-	-	-	0.99	-	-	0.11	-	-	-	-	-	-	-	0.12	0.97	-	-	0.77
E140	R144	-	-	1.00	1.00	-	0.70	-	1.00	1.00	1.00	-	-	1.00	1.00	-	1.00	1.00	-	-	-	1.00	-	-	1.00	-	1.00	-	1.00	-	1.00	-	1.00	1.00	1.00	1.00	1.00
**E140**	**R210**	-	-	0.93	1.00	-	1.00	-	1.00	1.00	1.00	-	-	1.00	1.00	-	1.00	1.00	-	-	-	1.00	-	-	1.00	-	1.00	-	1.00	-	0.77	-	1.00	1.00	0.95	1.00	1.00
**E140**	**R213**	-	-	1.00	-	-	0.95	-	-	-	0.50	-	-	-	0.91	-	-	-	-	-	-	-	-	-	-	-	1.00	-	-	-	0.36	-	-	-	1.00	0.97	-
F168	D172	-	-	-	-	-	-	-	-	0.96	0.29	-	-	-	0.12	-	-	-	-	-	-	-	-	-	-	-	0.45	-	-	-	0.43	-	-	0.94	-	-	-
C169	D172	-	1.00	-	-	-	0.20	-	-	-	0.64	-	0.32	-	0.59	-	-	-	0.88	-	-	-	0.93	-	-	-	0.52	-	-	-	0.46	-	-	-	0.85	-	-
I171	R210	-	-	0.72	-	-	-	-	-	-	-	-	-	-	-	0.69	-	-	-	0.25	-	-	-	-	-	-	-	0.13	-	-	-	-	-	-	-	0.20	-
D172	R144	-	-	-	-	-	-	-	-	-	-	-	1.00	-	0.12	-	-	-	-	-	-	-	0.19	0.28	-	-	-	-	-	-	0.95	-	-	-	-	0.15	-
S179	R207	0.55	-	-	-	-	-	-	-	-	-	-	-	-	-	-	-	-	-	-	-	-	-	0.23	0.12	-	-	0.19	-	-	-	-	-	-	-	0.91	-
S195	R198	-	-	0.54	-	0.26	-	0.31	-	-	-	-	-	-	-	-	-	0.76	-	-	-	-	-	-	-	0.40	-	-	-	0.24	-	-	-	-	-	-	-
L197	R201	-	-	0.93	-	-	-	-	-	-	-	-	-	-	-	-	-	0.20	-	-	1.00	-	-	-	-	0.23	-	-	-	0.21	-	-	-	-	-	-	-
Q204	R207	0.46	-	-	-	0.53	-	-	0.48	0.20	-	-	-	0.16	-	-	0.22	0.55	-	-	0.29	-	-	-	0.11	0.45	0.50	-	0.60	0.24	-	-	-	0.14	0.27	-	0.69
Q204	M208	0.17	-	-	-	-	-	-	0.45	0.32	-	-	0.80	0.14	-	-	0.31	0.26	-	-	0.14	-	-	-	0.33	0.35	0.70	-	0.61	0.29	-	-	-	0.54	0.91	-	0.61
L206	R210	-	-	-	-	-	-	-	-	-	-	-	0.83	-	-	-	-	-	-	-	-	-	-	-	-	-	0.41	-	0.53	-	-	-	-	0.18	0.93	-	0.76
R210	R213	-	0.13	0.28	-	-	-	0.48	-	-	-	0.52	-	-	-	-	-	-	0.61	-	-	-	0.54	-	-	-	0.45	-	-	-	0.26	0.69	-	-	0.17	0.61	0.31
M211	R213	-	-	0.16	-	-	-	0.34	-	-	-	0.12	-	-	-	-	-	-	0.16	-	-	-	-	-	-	-	0.13	-	-	-	0.18	-	-	-	0.17	-	0.16
D212	R213	-	1.00	0.58	-	-	-	0.29	-	-	-	0.62	-	-	-	1.00	-	-	0.34	1.00	-	-	-	1.00	-	-	0.38	-	-	-	0.52	-	0.19	1.00	1.00	0.56	0.15
R213	R87	-	-	0.13	-	-	-	-	-	-	-	-	-	-	-	0.70	-	-	-	-	-	-	-	0.12	-	-	-	-	-	-	-	-	-	-	-	0.19	0.12
# Activated				2	3		4		3	2	4			3	4		3	3				3			3		4		2		3		3	2	4	4	2
# Resting																																					

Table S2. List of interactions recorded within the VSD domains along each MD simulation of the apo KCNQ2 channel in the AC conformational state. Normalized contact frequencies are reported for each interaction pair having occupancy larger than 0.1 in at least one domain in one replica. Rows colored green and peach indicate interactions characterizing the active and resting states of the VSD domain, respectively (see also Table 1 and Figure 2). The last two rows summarize the number of such interactions simultaneously present in each domain along each MD simulation.

AO		REP1				REP2				REP3				REP4				REP5				REP6				REP7				REP8				REP9			
VSD domain		1	2	3	4	1	2	3	4	1	2	3	4	1	2	3	4	1	2	3	4	1	2	3	4	1	2	3	4	1	2	3	4	1	2	3	4
L81	E86	0.89	0.45	-	0.89	0.73	0.14	-	0.73	0.87	-	-	0.94	0.81	0.18	-	0.78	0.71	0.81	-	0.64	0.46	0.52	-	0.89	0.73	-	-	0.83	0.90	0.33	0.16	0.85	0.94	0.14	-	0.84
Y82	E86	-	-	0.59	-	-	0.49	0.84	-	-	0.66	0.88	-	-	0.19	0.90	-	-	-	1.00	-	0.18	0.16	0.94	-	-	0.58	0.94	-	-	0.14	0.64	-	-	0.53	0.76	-
E86	R87	0.34	-	0.40	0.46	0.14	-	-	0.46	-	-	-	-	0.27	-	-	0.52	0.44	-	-	0.32	-	-	-	0.21	0.24	-	-	0.16	0.23	-	-	0.24	0.23	-	-	0.30
E86	R144	1.00	-	1.00	1.00	1.00	1.00	1.00	1.00	-	-	1.00	-	1.00	1.00	-	1.00	1.00	0.89	-	1.00	-	-	1.00	0.99	1.00	-	-	1.00	1.00	-	-	1.00	1.00	1.00	-	1.00
S110	R201	-	-	0.27	0.51	-	-	-	-	-	-	-	-	-	-	-	0.28	-	-	-	-	-	-	0.31	-	-	-	-	-	-	-	-	-	-	-	-	0.57
S110	Q204	-	-	-	-	-	-	-	-	-	-	0.16	-	-	-	0.43	0.11	-	-	-	-	-	-	0.37	-	-	-	-	-	-	-	0.38	-	-	-	-	-
S113	R201	-	-	0.37	0.35	-	-	0.70	0.12	-	0.20	0.55	-	-	-	0.80	0.15	-	-	0.83	0.11	-	-	-	0.12	-	-	0.35	0.15	-	-	0.60	0.16	-	-	0.19	0.19
E130	I134	-	-	-	0.98	0.90	0.64	-	-	-	0.94	-	0.99	-	0.66	-	-	0.97	0.62	-	-	-	0.89	-	-	0.92	-	-	-	-	0.95	-	-	-	-	0.96	1.00
E130	S179	-	-	-	1.00	1.00	-	-	-	-	0.82	-	0.84	-	0.83	-	-	0.92	0.37	-	-	-	-	-	-	0.93	-	-	-	-	0.90	-	-	-	-	1.00	0.99
**E130**	**Q204**	-	-	-	0.87	0.77	1.00	-	-	-	0.91	-	0.95	-	0.93	-	-	0.43	0.68	-	-	-	0.53	-	-	0.84	-	-	-	-	0.18	-	-	-	-	0.33	0.97
**E130**	**R207**	-	-	-	1.00	1.00	1.00	-	-	-	1.00	-	1.00	-	1.00	-	-	1.00	1.00	-	-	-	1.00	-	-	1.00	-	-	-	-	1.00	-	-	-	-	1.00	1.00
T133	F137	-	-	-	0.99	0.21	0.91	-	-	-	0.94	-	0.95	-	0.94	-	-	0.95	0.95	-	-	-	0.93	-	-	0.64	-	-	-	-	0.94	-	-	-	-	0.97	0.99
V135	E140	-	-	-	-	-	0.88	-	-	-	0.21	-	-	-	0.20	-	-	-	0.95	-	-	-	0.20	-	-	-	-	-	-	-	0.60	-	-	-	0.40	0.42	-
V136	E140	-	-	-	0.88	0.94	-	-	-	-	0.76	-	0.81	-	0.14	-	-	0.45	-	-	-	-	0.41	-	-	0.85	-	-	-	-	-	-	-	-	0.25	0.25	0.88
V136	R210	-	-	-	-	-	0.16	-	-	-	0.14	-	-	-	-	-	-	-	0.70	-	-	-	0.16	-	-	-	-	-	-	-	0.13	-	-	-	0.35	-	-
F137	Y141	-	-	-	0.94	0.71	0.55	-	-	-	0.89	-	0.92	-	0.36	-	-	0.94	0.31	-	-	-	0.88	-	-	0.72	-	-	-	-	0.81	-	-	-	0.70	0.54	0.93
E140	Y95	-	-	-	-	0.35	0.24	-	-	-	0.99	-	-	-	0.17	-	-	-	0.97	-	-	-	0.53	-	-	0.62	-	-	-	-	0.20	-	-	-	0.50	-	-
E140	R144	-	-	-	1.00	1.00	1.00	-	-	-	1.00	-	1.00	-	1.00	-	-	1.00	1.00	-	-	-	1.00	-	-	1.00	-	-	-	-	1.00	-	-	-	1.00	1.00	1.00
**E140**	**R210**	-	-	-	1.00	1.00	1.00	-	-	-	1.00	-	1.00	-	1.00	-	-	1.00	0.86	-	-	-	1.00	-	-	1.00	-	-	-	-	1.00	-	-	-	1.00	1.00	1.00
**E140**	**R213**	-	-	-	1.00	-	1.00	-	-	-	1.00	-	1.00	-	1.00	-	-	-	1.00	-	-	-	1.00	-	-	-	-	-	-	-	0.63	-	-	-	1.00	0.22	1.00
F168	D172	-	-	-	-	-	-	0.72	-	-	-	0.17	-	-	-	-	-	-	-	0.58	-	-	0.67	-	-	-	-	-	-	-	-	-	-	-	-	0.90	-
C169	D172	-	-	-	-	0.98	-	-	-	0.89	-	0.58	-	0.75	-	-	-	0.30	-	-	-	0.67	-	0.20	-	0.78	-	-	-	-	-	0.39	-	0.91	0.14	-	-
D172	R144	1.00	-	0.86	1.00	1.00	-	1.00	-	1.00	-	1.00	-	1.00	-	0.15	-	1.00	-	0.80	-	1.00	-	0.86	-	1.00	-	1.00	-	1.00	-	1.00	-	1.00	1.00	1.00	-
S179	Q204	0.39	0.98	-	-	-	0.97	-	-	-	-	-	-	-	-	-	-	-	0.72	-	-	-	0.74	-	-	0.13	0.97	-	-	-	-	-	-	-	-	-	-
S195	R198	-	-	0.22	-	-	-	-	-	-	-	0.47	0.30	-	-	-	-	-	-	0.41	-	-	-	-	-	-	-	-	-	-	-	0.26	-	-	-	-	0.28
L197	R201	-	-	0.43	-	-	-	0.41	-	-	-	0.80	0.71	-	-	-	-	-	-	0.94	-	-	-	-	-	-	-	-	-	-	-	0.62	-	-	-	-	0.82
R198	R201	-	-	-	-	-	0.56	-	-	-	-	-	-	-	0.29	-	-	-	0.91	-	-	-	-	-	-	-	0.45	-	-	-	-	-	-	-	0.77	-	-
R198	F202	-	-	-	-	0.58	-	-	-	0.57	-	-	-	-	-	-	-	0.47	-	-	-	0.56	0.13	-	-	-	-	-	-	-	0.46	-	-	0.59	-	-	-
L200	Q204	-	-	-	-	0.40	-	-	-	0.71	-	-	-	-	0.34	-	-	-	-	-	-	-	0.44	-	-	-	0.81	-	-	-	0.80	-	-	-	-	-	-
R201	*Q204*	-	-	-	0.41	-	-	0.98	0.38	-	0.12	-	0.54	-	0.17	-	-	-	-	-	-	-	-	-	-	-	-	-	-	-	-	-	-	-	-	0.47	-
R201	I205	-	-	-	0.43	-	-	0.35	0.76	-	0.67	-	0.77	-	0.88	-	-	-	-	-	-	-	-	-	-	-	-	0.78	-	-	-	-	-	-	-	0.53	-
L203	R207	-	-	-	0.28	-	-	0.18	0.38	-	0.61	-	0.18	-	-	-	-	-	-	-	-	-	-	-	-	-	-	0.38	-	-	-	-	-	-	-	0.28	-
Q204	R207	0.85	-	1.00	0.57	-	0.95	0.60	0.35	0.87	-	1.00	0.70	-	-	1.00	0.71	0.82	-	1.00	0.65	-	1.00	1.00	0.74	0.91	0.96	0.68	1.00	0.58	1.00	1.00	1.00	0.86	1.00	1.00	1.00
L206	R210	0.32	-	0.48	-	-	-	-	-	0.90	-	0.37	-	-	-	0.68	0.11	0.34	-	-	-	-	0.78	0.28	-	0.76	0.21	-	-	0.37	0.26	0.15	0.45	0.91	-	-	-
# Activated					4	3	4				4		4		4			2	4				4			3					3				2	2	4
# Resting								1					1																								

Table S3. List of interactions recorded within the VSD domains along each MD simulation of the apo KCNQ2 channel in the AO conformational state. See the caption of Table S2 for further details.

RC		REP1				REP2				REP3				REP4				REP5				REP6				REP7				REP8				REP9			
VSD domain		1	2	3	4	1	2	3	4	1	2	3	4	1	2	3	4	1	2	3	4	1	2	3	4	1	2	3	4	1	2	3	4	1	2	3	4
Y82	E86	0.95	0.74	0.89	0.91	0.34	0.54	0.85	0.81	0.27	0.75	0.88	0.59	0.29	0.66	0.91	0.62	0.25	0.90	0.93	0.41	1.00	0.77	0.94	0.68	0.53	0.90	0.91	0.75	0.44	0.88	0.91	0.56	0.22	0.87	0.92	0.65
N83	E86	-	-	-	-	-	-	-	-	0.59	-	-	-	0.68	-	-	-	0.61	-	-	-	-	-	-	-	0.36	-	-	-	-	-	-	0.39	0.63	-	-	-
E86	R87	-	-	-	-	-	0.62	1.00	-	-	-	1.00	-	-	-	0.43	-	-	0.43	0.13	-	-	0.23	-	-	-	0.49	0.41	-	-	-	-	-	-	-	0.37	-
E86	R144	-	-	-	-	-	1.00	0.13	-	-	-	1.00	-	-	1.00	0.92	-	-	0.25	1.00	-	-	0.91	0.59	-	-	0.39	1.00	-	-	-	1.00	-	-	-	0.33	-
E86	*R207*	-	-	-	-	-	0.15	1.00	-	-	-	1.00	-	-	1.00	1.00	-	-	0.88	1.00	-	-	0.13	1.00	-	-	-	1.00	-	-	-	1.00	-	-	-	1.00	-
E130	I134	-	-	-	-	-	-	-	0.74	-	-	-	0.97	0.96	-	-	0.75	-	-	0.96	0.83	-	0.99	-	0.95	-	-	-	0.94	-	-	0.96	-	-	-	-	0.98
E130	S179	-	-	-	-	-	-	-	1.00	-	-	-	0.98	1.00	-	-	1.00	-	-	0.92	1.00	-	0.84	-	1.00	-	-	-	0.93	-	-	1.00	-	-	-	-	1.00
E130	N190	-	-	-	-	-	-	-	0.24	-	-	-	0.82	-	-	-	-	-	-	-	0.17	-	0.49	-	0.54	-	-	-	0.59	-	-	-	-	-	-	-	0.82
E130	S195	-	-	-	-	-	-	-	-	-	-	-	0.20	-	-	-	0.99	-	-	0.99	0.81	-	0.59	-	0.42	-	-	-	0.15	-	-	-	-	-	-	-	0.65
E130	*R198*	-	-	-	-	-	-	-	0.89	-	-	-	1.00	0.89	-	-	1.00	-	-	0.95	0.86	-	1.00	-	0.83	-	-	-	1.00	-	-	0.86	-	-	-	-	1.00
V132	F137	-	-	-	-	-	-	-	0.81	-	-	-	0.22	0.96	-	-	0.69	-	-	-	0.68	-	-	-	0.51	-	-	-	0.35	-	-	-	-	-	-	-	0.66
T133	F137	-	-	-	-	-	-	-	-	-	-	-	0.63	-	-	-	-	-	-	1.00	-	-	0.90	-	0.13	-	-	-	0.17	-	-	0.93	-	-	-	-	-
V136	E140	-	-	-	-	-	-	-	0.88	-	-	-	0.49	0.98	-	-	0.88	-	-	0.94	0.78	-	0.93	-	0.83	-	-	-	0.88	-	-	0.49	-	-	-	-	0.78
F137	Y141	-	-	-	-	-	-	-	0.99	-	-	-	0.82	0.99	-	-	0.97	-	-	0.96	0.99	-	0.95	-	0.98	-	-	-	0.99	-	-	0.95	-	-	-	-	0.97
E140	Y95	-	-	-	-	-	-	-	1.00	-	-	-	-	1.00	-	-	0.92	-	-	0.98	0.96	-	1.00	-	0.99	-	-	-	0.87	-	-	0.70	-	-	-	-	0.99
E140	R144	-	-	-	-	-	-	-	0.72	-	-	-	-	0.86	-	-	1.00	-	-	1.00	0.53	-	1.00	-	1.00	-	-	-	1.00	-	-	1.00	-	-	-	-	0.71
E140	*R201*	-	-	-	-	-	-	-	1.00	-	-	-	-	1.00	-	-	1.00	-	-	1.00	1.00	-	1.00	-	1.00	-	-	-	1.00	-	-	1.00	-	-	-	-	1.00
E140	R207	-	-	-	-	-	-	-	1.00	-	-	-	-	-	-	-	0.12	-	-	-	1.00	-	-	-	0.16	-	-	-	0.39	-	-	-	-	-	-	-	1.00
Y141	R201	0.45	-	-	0.12	-	-	-	-	0.33	-	-	-	0.21	-	-	-	0.26	-	0.13	-	0.37	-	-	-	0.31	-	-	-	0.42	-	-	-	-	-	-	-
Y141	Q204	-	-	-	0.33	-	-	-	1.00	-	-	-	1.00	-	-	-	0.46	-	-	-	1.00	0.31	-	-	0.56	0.22	-	-	0.47	-	-	-	0.92	-	-	-	1.00
C169	D172	0.33	-	-	0.13	0.14	-	-	-	-	-	-	-	0.71	-	-	-	0.12	-	-	0.16	0.11	-	-	0.11	-	-	-	-	0.22	-	-	-	-	-	-	-
I171	Q204	-	-	-	0.11	0.11	-	-	-	-	-	-	-	0.34	-	-	-	0.14	-	-	0.77	0.18	-	-	0.23	-	-	-	0.28	0.21	-	-	-	-	-	-	-
D172	R144	1.00	-	-	1.00	1.00	-	-	-	-	-	-	-	1.00	-	-	1.00	1.00	-	-	1.00	1.00	-	-	1.00	1.00	-	-	1.00	1.00	-	-	-	-	-	-	-
T194	R198	-	-	-	-	-	0.20	-	-	-	0.27	-	-	-	0.17	-	0.90	-	-	0.25	-	-	0.61	-	-	-	0.16	-	-	-	-	-	-	-	-	-	-
R201	*Q204*	-	-	1.00	0.32	-	-	1.00	0.85	-	-	1.00	0.98	-	-	1.00	0.25	-	-	1.00	0.47	-	-	1.00	0.76	-	-	0.96	0.75	-	0.30	1.00	0.88	0.34	-	0.95	0.87
R201	I205	-	-	0.82	-	-	-	0.73	-	-	-	0.25	0.53	-	-	0.53	0.69	-	-	0.52	-	-	-	0.54	0.19	-	-	0.74	0.14	-	0.71	0.17	-	0.52	-	0.84	-
L203	R207	-	-	0.45	0.28	-	-	0.28	-	-	-	0.33	-	-	-	0.57	-	-	-	0.41	-	-	-	0.71	0.58	-	-	0.47	0.23	-	0.48	0.53	0.54	0.73	-	0.64	0.13
Q204	M208	-	-	0.84	-	-	-	0.59	-	-	-	1.00	-	-	-	0.99	-	-	-	0.72	-	-	-	0.64	-	-	-	0.87	-	-	-	0.88	-	-	-	0.78	-
L206	R210	-	-	0.17	-	-	-	-	-	-	-	0.19	-	-	-	0.30	0.95	-	-	0.23	-	-	-	0.24	-	-	-	-	-	-	-	-	-	-	-	0.28	-
R207	R210	-	-	-	-	-	0.46	0.23	-	-	-	-	-	-	-	-	-	-	-	-	-	-	-	0.24	-	-	-	-	-	-	0.85	-	-	-	-	0.31	-
# Activated																																					
# Resting				1				2	3			2	2	2	1	2	2		1	4	2		2	2	3			2	3			4	1			2	3

Table S4. List of interactions recorded within the VSD domains along each MD simulation of the apo KCNQ2 channel in the RC conformational state. See the caption of Table S2 for further details.

		Channel state		
		AC	AO	RC
RTG site	1	4	14	6
	2	5	8	1
	3	7	10	4
	4	7	10	5
	Sum	23	42	16
	Protein	27	32	14

Table S5. Number of conformational clusters calculated at each of the four RTG binding sites and on the whole protein from the cumulative trajectory of the apo KCNQ2 tetramer. The cluster analyses were performed on the non-hydrogenous and C_α atoms for the RTG binding site and the whole protein, respectively.

BS-RTG		1															
SIMULATION		1				2				4				5			
VSD domain		1	2	3	4	1	2	3	4	1	2	3	4	1	2	3	4
L81	E86	-	0.66	-	-	-	-	-	-	-	0.59	-	-	-	0.28	-	-
Y82	E86	0.83	-	0.71	0.94	0.77	0.52	0.75	0.85	-	-	0.91	0.73	0.51	-	0.81	0.41
L85	R213	-	-	-	0.95	-	-	-	-	-	-	-	-	-	0.25	-	-
E86	R87	-	0.22	0.19	-	-	-	-	-	-	-	-	-	0.12	-	-	-
E86	R144	1.00	1.00	1.00	-	1.00	-	1.00	1.00	-	0.93	1.00	-	0.94	0.86	1.00	1.00
E86	K166	-	-	0.16	-	-	-	-	0.37	-	-	-	-	-	-	-	-
E86	R213	1.00	-	1.00	0.95	-	-	-	-	-	-	1.00	-	0.64	-	-	-
S110	Q204	-	-	-	-	-	-	-	-	-	-	-	-	-	-	-	-
E130	I134	-	-	0.95	0.98	-	0.97	-	0.99	-	-	-	0.98	-	0.93	-	0.98
E130	S179	-	-	0.96	0.97	-	-	-	0.94	-	-	-	0.92	-	-	-	0.98
**E130**	**Q204**	-	-	0.99	0.53	-	0.99	-	0.96	-	-	-	0.96	-	1.00	-	0.87
**E130**	**R207**	-	-	1.00	1.00	-	1.00	-	1.00	-	-	-	1.00	-	1.00	-	1.00
T133	F137	-	-	0.96	0.95	-	-	-	0.97	-	-	-	0.98	-	0.82	-	0.99
T133	R207	-	-	-	-	-	-	-	-	-	-	-	-	-	-	-	-
V136	E140	-	-	0.68	0.93	-	-	-	0.94	-	-	-	0.89	-	0.29	-	0.98
F137	Y141	-	-	0.93	0.55	-	-	-	0.90	-	-	-	0.94	-	0.42	-	0.87
E140	Y95	-	-	0.17	-	-	-	-	0.90	-	-	-	0.90	-	0.38	-	-
E140	R144	-	-	1.00	1.00	-	-	-	1.00	-	-	-	1.00	-	1.00	-	1.00
**E140**	**R210**	-	-	1.00	-	-	-	-	1.00	-	-	-	1.00	-	1.00	-	1.00
**E140**	**R213**	-	-	-	-	-	-	-	-	-	-	-	-	-	1.00	-	-
F168	D172	-	-	-	-	-	-	-	-	-	-	-	-	-	-	-	-
C169	D172	-	-	-	-	-	-	-	-	-	0.43	-	-	-	-	-	-
D172	R144	-	-	1.00	-	-	0.92	-	-	-	1.00	-	-	-	-	-	-
S179	Q204	0.15	-	-	-	-	0.90	-	-	-	-	-	-	-	0.96	-	-
L197	R201	-	0.84	-	-	-	-	-	-	-	0.95	-	-	-	-	-	-
R198	R201	-	-	-	0.34	-	-	-	-	-	-	-	0.27	-	0.33	-	-
L200	Q204	-	0.74	-	-	-	-	-	-	-	-	-	0.12	-	0.33	-	-
R201	*Q204*	-	0.16	1.00	-	-	-	1.00	1.00	-	-	1.00	1.00	0.14	-	1.00	-
R201	I205	-	-	0.61	-	-	-	0.85	0.87	-	-	0.44	0.70	-	0.89	0.80	-
L203	R207	-	-	0.58	-	-	-	0.16	0.13	-	-	0.26	-	-	-	0.13	-
Q204	R207	0.66	-	-	0.84	0.89	-	-	-	0.87	0.86	-	-	1.00	0.59	-	0.24
Q204	M208	0.17	-	-	0.59	-	-	-	-	-	-	-	-	-	-	-	-
L206	R210	0.91	-	-	-	-	-	-	-	0.20	-	-	-	0.75	-	-	-
# Activated				3	2		2		3				3		4		3
# Resting				1				1	1			1	1			1	

BS-RTG		2											
SIMULATION		1				2				3			
VSD domain		1	2	3	4	1	2	3	4	1	2	3	4
L81	E86	-	-	-	-	-	-	-	-	-	-	-	0.96
Y82	E86	0.59	0.75	0.65	-	0.81	0.61	0.34	0.44	0.86	0.85	0.47	-
L85	R213	-	-	-	-	-	-	-	-	-	-	-	-
E86	R87	-	-	-	0.24	-	-	-	-	-	-	-	0.52
E86	R144	-	1.00	1.00	1.00	-	0.32	-	-	-	0.34	-	1.00
E86	K166	-	0.37	-	0.22	-	-	-	-	-	-	-	-
E86	R213	-	1.00	-	-	-	-	-	-	-	-	-	-
S110	Q204	0.22	-	-	-	-	-	-	0.46	0.23	-	-	-
E130	I134	0.89	-	-	0.96	0.96	0.95	-	0.99	-	0.92	0.99	-
E130	S179	0.18	-	-	0.97	1.00	0.98	-	0.99	-	0.49	0.98	-
**E130**	**Q204**	0.19	-	-	0.96	0.74	0.97	-	-	-	0.25	0.98	-
**E130**	**R207**	1.00	-	-	1.00	1.00	1.00	-	1.00	-	1.00	1.00	-
T133	F137	0.89	-	-	0.98	0.81	0.87	-	1.00	-	0.87	0.99	-
T133	R207	0.14	-	-	-	0.74	0.51	-	-	-	0.34	-	-
V136	E140	0.74	-	-	0.90	0.78	0.98	-	0.96	-	0.13	0.98	-
F137	Y141	0.86	-	-	0.89	0.86	0.92	-	0.96	-	0.89	0.85	-
E140	Y95	-	-	-	-	-	0.94	-	-	-	0.92	1.00	-
E140	R144	1.00	-	-	1.00	1.00	1.00	-	1.00	-	1.00	1.00	-
**E140**	**R210**	1.00	-	-	1.00	1.00	1.00	-	1.00	-	0.34	-	-
**E140**	**R213**	-	-	-	-	-	1.00	-	-	-	1.00	1.00	-
F168	D172	-	-	-	-	-	-	0.96	0.62	-	-	-	-
C169	D172	0.11	-	-	-	-	-	-	-	0.30	-	-	-
D172	R144	1.00	1.00	-	-	1.00	-	-	-	1.00	-	-	-
S179	Q204	0.23	-	-	-	-	-	-	-	-	0.43	-	-
L197	R201	-	-	-	-	-	0.91	0.91	-	-	-	-	-
R198	R201	-	0.19	-	0.11	-	-	-	-	0.46	-	-	0.38
L200	Q204	-	-	-	-	-	-	-	-	-	-	-	-
R201	*Q204*	-	0.30	1.00	1.00	-	-	1.00	-	-	-	1.00	-
R201	I205	-	0.54	0.55	0.96	-	-	0.80	-	-	-	0.79	-
L203	R207	-	0.62	0.56	-	-	-	0.35	-	-	-	0.54	-
Q204	R207	0.98	-	-	-	0.88	0.81	-	-	1.00	0.51	-	-
Q204	M208	-	-	-	-	-	-	-	-	0.47	0.44	-	-
L206	R210	0.84	-	-	-	0.91	0.73	-	-	0.87	0.64	-	-
# Activated		2			3	3	4		2		2	3	
# Resting				1	1			1				1	

BS-RTG		3											
SIMULATION		1				2				3			
VSD domain		1	2	3	4	1	2	3	4	1	2	3	4
L81	E86	-	-	-	-	-	-	-	0.30	0.35	-	-	-
Y82	E86	0.70	0.79	-	0.92	0.75	0.55	0.69	0.28	-	0.87	0.88	0.91
L85	R213	-	0.27	-	-	-	0.76	-	-	-	0.88	-	-
E86	R87	0.16	-	-	-	-	-	-	-	-	-	-	-
E86	R144	1.00	1.00	0.42	-	-	1.00	1.00	-	1.00	1.00	-	-
E86	K166	-	-	-	-	-	-	0.38	-	-	0.90	-	-
E86	R213	-	-	1.00	-	-	-	0.95	-	-	-	-	-
S110	Q204	-	-	0.74	-	-	-	-	-	-	-	-	-
E130	I134	-	0.95	-	-	0.96	-	0.99	0.98	-	0.93	-	-
E130	S179	-	-	-	-	0.98	-	0.99	0.35	-	-	-	-
**E130**	**Q204**	-	1.00	-	-	0.71	-	0.93	0.98	-	0.96	-	-
**E130**	**R207**	-	1.00	-	-	1.00	-	1.00	1.00	-	1.00	-	-
T133	F137	-	0.96	-	-	0.96	-	0.96	0.97	-	0.99	-	-
T133	R207	-	-	-	-	0.72	-	-	-	-	-	-	-
V136	E140	-	0.92	-	-	0.61	-	0.42	0.83	-	0.90	-	-
F137	Y141	-	0.73	-	-	0.96	-	0.81	0.88	-	0.87	-	-
E140	Y95	-	0.61	-	-	0.96	-	-	-	-	-	-	-
E140	R144	-	1.00	-	-	1.00	-	1.00	1.00	-	1.00	-	-
**E140**	**R210**	-	1.00	-	-	1.00	-	1.00	1.00	-	1.00	-	-
**E140**	**R213**	-	1.00	-	-	-	-	0.33	-	-	0.80	-	-
F168	D172	-	-	-	-	-	-	0.72	-	-	-	-	-
C169	D172	-	-	-	-	-	-	-	-	-	-	-	-
D172	R144	1.00	-	-	1.00	-	-	-	-	-	1.00	-	-
S179	Q204	-	0.93	-	-	-	-	-	-	-	-	-	-
L197	R201	0.92	0.90	-	-	-	-	-	0.46	-	-	-	-
R198	R201	-	-	-	0.34	-	-	-	0.22	-	-	0.36	-
L200	Q204	-	-	-	0.31	-	-	-	-	-	0.74	-	-
R201	*Q204*	-	-	-	0.22	-	-	0.83	0.52	-	-	1.00	1.00
R201	I205	-	-	-	0.94	-	-	0.49	0.41	-	-	0.85	0.82
L203	R207	-	-	-	-	-	-	0.89	0.49	-	-	0.43	0.18
Q204	R207	-	0.72	-	-	-	0.93	-	-	-	-	-	-
Q204	M208	-	-	-	-	-	-	-	-	-	-	-	-
L206	R210	-	-	-	-	-	-	-	-	-	-	-	-
# Activated			4			3		3	3		4		
# Resting								1	1			1	1

BS-RTG		4							
SIMULATION		1				2			
VSD domain		1	2	3	4	1	2	3	4
L81	E86	-	-	-	-	0.24	-	-	-
Y82	E86	0.97	0.80	0.91	0.88	-	0.79	0.54	0.72
L85	R213	-	-	-	0.90	-	-	-	0.48
E86	R87	-	-	-	-	0.15	-	-	-
E86	R144	-	-	-	-	0.38	0.35	1.00	1.00
E86	K166	-	-	-	-	-	-	-	-
E86	R213	-	-	-	-	1.00	0.79	-	-
S110	Q204	0.41	-	-	-	-	-	0.24	-
E130	I134	0.93	0.95	0.94	0.99	-	0.98	1.00	0.96
E130	S179	-	-	0.90	-	-	0.13	0.83	0.99
**E130**	**Q204**	0.67	1.00	0.96	0.78	-	0.64	-	0.99
**E130**	**R207**	1.00	1.00	1.00	1.00	-	1.00	1.00	1.00
T133	F137	-	0.94	1.00	0.95	-	0.80	0.95	0.43
T133	R207	-	-	-	-	-	0.24	-	0.37
V136	E140	-	0.86	0.89	0.94	-	0.90	0.96	0.88
F137	Y141	-	0.99	0.91	0.86	-	0.85	0.82	0.70
E140	Y95	-	0.96	-	0.53	-	0.29	0.83	0.96
E140	R144	-	1.00	0.83	1.00	-	1.00	1.00	1.00
**E140**	**R210**	-	1.00	0.52	1.00	-	1.00	1.00	-
**E140**	**R213**	-	-	1.00	1.00	-	-	1.00	1.00
F168	D172	-	0.79	-	-	-	-	-	-
C169	D172	-	-	-	-	-	-	0.30	-
D172	R144	-	-	-	-	-	1.00	0.56	-
S179	Q204	-	0.86	-	-	-	0.62	-	-
L197	R201	-	-	-	-	-	-	-	-
R198	R201	-	0.21	0.42	0.50	-	-	-	-
L200	Q204	-	0.27	-	-	-	-	-	-
R201	*Q204*	-	-	0.88	0.79	-	-	-	1.00
R201	I205	-	-	0.71	0.46	-	-	-	-
L203	R207	-	-	-	-	-	-	-	0.24
Q204	R207	0.76	0.47	-	0.21	0.88	0.96	0.80	-
Q204	M208	0.54	-	-	-	-	-	0.90	-
L206	R210	0.18	-	-	-	-	-	0.96	-
# Activated		2	3	4	4		3	3	3
# Resting				1	1				1

Table S6. List of interactions recorded within the VSD domains along different MD simulations of the complex between one RTG molecule and the KCNQ2 channel in the AO conformational state. See caption of Table S2 for further details.

BS-RTG		1																							
SIMULATION		1				2				3				4				5				6			
VSD domain		1	2	3	4	1	2	3	4	1	2	3	4	1	2	3	4	1	2	3	4	1	2	3	4
Y82	E86	1.00	0.82	0.67	0.70	1.00	0.88	0.84	0.24	0.89	0.88	0.85	0.37	0.81	0.88	0.81	0.51	0.24	0.75	0.88	0.77	0.13	0.94	0.93	0.27
E86	R87	-	-	-	-	-	1.00	0.30	-	-	-	-	-	-	-	-	-	-	-	-	-	-	1.00	-	-
E86	R144	-	0.33	0.41	-	-	0.22	-	-	-	-	1.00	-	-	-	0.13	-	-	-	-	-	-	0.39	-	-
E86	R207	-	1.00	1.00	-	-	1.00	1.00	-	-	-	-	-	-	-	1.00	-	-	-	1.00	-	-	1.00	-	-
E130	I134	-	-	-	-	-	-	0.77	-	-	-	-	-	0.98	-	-	-	0.88	0.92	-	-	0.56	-	-	-
E130	S179	-	-	-	-	-	-	-	-	-	-	-	-	0.22	-	-	-	-	1.00	-	-	-	-	-	-
E130	T194	-	-	-	-	-	-	-	-	-	-	-	-	0.12	-	-	-	-	-	-	-	-	-	-	-
E130	R198	-	-	-	-	-	-	1.00	-	-	-	-	-	1.00	-	-	-	0.91	1.00	-	-	1.00	-	-	-
V132	F137	-	-	-	-	-	-	-	-	-	-	-	-	-	-	-	-	0.78	0.27	-	-	0.39	-	-	-
T133	F137	-	-	-	-	-	-	0.98	-	-	-	-	-	0.99	-	-	-	-	-	-	-	0.13	-	-	-
T133	R198	-	-	-	-	-	0.13	0.66	-	-	-	-	-	-	-	-	-	0.19	0.93	-	-	0.31	-	-	-
V136	E140	-	-	-	-	-	0.65	0.68	-	-	-	-	-	0.96	-	-	-	0.94	0.96	-	-	0.88	-	-	-
F137	Y141	-	-	-	-	-	0.99	0.98	-	-	-	-	-	0.94	-	-	-	0.87	0.91	-	-	1.00	-	-	-
E140	Y95	-	-	-	-	-	0.18	0.98	-	-	-	-	-	0.96	-	-	-	1.00	0.91	-	-	0.99	-	-	-
E140	R144	-	-	-	-	-	1.00	1.00	-	-	-	-	-	1.00	-	-	-	0.56	1.00	-	-	0.98	-	-	-
E140	R201	-	-	-	-	-	1.00	1.00	-	-	-	-	-	1.00	-	-	-	1.00	1.00	-	-	1.00	-	-	-
Y141	Q204	0.78	-	-	-	-	-	-	1.00	-	-	-	0.29	-	-	-	-	-	-	-	-	-	-	-	0.91
T194	R198	-	-	0.98	1.00	0.98	-	-	-	-	-	-	-	-	-	-	0.95	-	-	0.97	1.00	-	1.00	0.92	0.87
R201	Q204	0.26	-	1.00	1.00	0.92	0.18	0.73	-	-	-	0.76	0.99	-	-	1.00	-	1.00	0.19	-	1.00	-	-	0.32	-
R201	I205	0.68	-	0.19	-	-	0.54	0.81	-	-	-	0.81	0.87	-	-	0.25	-	-	0.97	-	-	-	-	0.80	0.83
L203	R207	-	-	0.66	-	0.25	0.84	0.74	-	-	-	0.50	-	-	-	0.44	-	0.92	0.19	-	-	-	-	0.92	0.18
Q204	R207	-	-	-	-	-	0.91	0.27	-	-	-	0.22	-	-	-	-	-	-	-	-	-	-	1.00	0.55	-
Q204	M208	-	-	-	-	-	0.91	0.69	-	-	-	0.72	-	-	-	0.38	-	-	-	-	-	-	0.78	0.84	-
L206	R210	-	-	-	-	-	0.12	0.76	-	-	-	0.22	-	-	-	0.48	-	-	-	-	-	-	0.48	0.93	-
R207	M211	-	-	-	-	-	-	-	-	-	-	-	-	0.99	-	-	-	0.99	-	-	-	-	-	-	-
# Activated																									
# Resting			1	2	1	1	2	4				1	1	2		2		3	2	1	1	2	1		

BS-RTG		2															
SIMULATION		1				2				3				4			
VSD domain		1	2	3	4	1	2	3	4								
Y82	E86	-	0.90	0.83	0.93	0.51	0.89	0.86	0.70	0.52	0.94	0.82	0.71	0.63	0.78	0.87	0.74
E86	R87	-	0.80	-	-	-	0.45	0.14	-	-	-	0.88	0.48	0.99	1.00	-	-
E86	R144	1.00	1.00	-	-	-	1.00	0.82	1.00	-	-	0.52	-	0.83	1.00	0.99	0.70
E86	R207	0.42	-	-	-	-	-	1.00	1.00	-	-	1.00	1.00	1.00	1.00	1.00	1.00
E130	I134	-	-	-	-	-	-	-	0.84	0.92	-	0.94	-	0.99	0.96	-	-
E130	S179	-	-	-	-	-	-	-	0.99	1.00	-	1.00	-	0.97	0.41	-	-
E130	T194	-	-	-	-	-	-	-	0.99	-	-	1.00	-	-	-	-	-
E130	R198	-	-	-	-	-	-	-	1.00	0.63	-	1.00	-	1.00	1.00	-	-
V132	F137	-	-	-	-	-	-	-	0.53	0.53	-	-	-	-	-	-	-
T133	F137	-	-	-	-	-	-	-	-	-	-	0.97	-	0.97	0.98	-	-
T133	R198	-	-	-	-	-	-	-	0.55	0.65	-	-	-	-	-	-	-
V136	E140	-	-	-	-	-	-	-	0.97	0.96	-	0.58	-	0.96	0.45	-	-
F137	Y141	-	-	-	-	-	-	-	0.84	1.00	-	0.98	-	0.98	0.99	-	-
E140	Y95	-	-	-	-	-	-	-	0.85	1.00	-	0.65	-	0.93	0.20	-	-
E140	R144	-	-	-	-	-	-	-	1.00	1.00	-	1.00	-	1.00	1.00	-	-
E140	R201	-	-	-	-	-	-	-	-	1.00	-	1.00	-	1.00	1.00	-	-
Y141	Q204	-	-	-	-	-	-	-	-	-	-	-	-	-	-	-	-
T194	R198	0.94	-	0.80	-	-	-	-	-	-	-	-	-	-	-	-	1.00
R201	Q204	1.00	0.25	-	0.34	1.00	0.36	0.19	1.00	-	0.62	0.14	0.84	-	0.84	0.55	1.00
R201	I205	0.69	0.87	-	0.71	0.27	0.95	0.74	-	-	0.39	0.75	-	-	0.43	0.95	-
L203	R207	0.75	0.95	-	0.77	0.81	0.78	0.63	-	-	0.50	0.86	-	-	0.83	0.50	0.18
Q204	R207	-	-	-	-	-	-	0.75	0.71	-	-	0.56	1.00	1.00	-	-	0.50
Q204	M208	-	0.94	-	-	-	0.89	0.79	0.80	-	0.96	0.81	0.79	0.60	0.89	0.81	0.23
L206	R210	-	0.97	-	-	-	0.94	0.32	0.29					0.84	0.93	0.37	0.90
R207	M211	0.85	0.48	-	-	-	0.46	-	-					-	0.61	0.86	-
# Activated																	
# Resting		1				1		1	3	2	1	3	2	3	4	2	2

continued

BS-RTG		3																			
SIMULATION		1				2				3				4				5			
VSD domain		1	2	3	4	1	2	3	4	1	2	3	4	1	2	3	4	1	2	3	4
Y82	E86	0.86	0.91	0.93	-	0.58	0.92	0.84	0.34	0.61	0.94	0.78	0.59	1.00	0.82	0.83	0.72	0.50	0.78	0.79	0.28
E86	R87	1.00	0.89	-	-	-	0.71	0.12	-	-	1.00	0.25	-	-	0.57	-	-	-	-	0.28	-
E86	R144	-	0.93	1.00	-	-	0.20	-	-	-	0.54	0.54	-	-	1.00	0.68	-	-	-	0.61	-
E86	R207	1.00	1.00	1.00	-	-	1.00	1.00	-	-	-	1.00	-	-	-	1.00	-	-	-	1.00	-
E130	I134	-	0.82	-	-	-	-	-	-	0.84	-	-	0.96	0.94	0.95	-	-	-	0.98	-	0.78
E130	S179	-	-	-	-	-	-	-	-	0.95	-	-	1.00	0.99	-	-	-	-	-	-	0.84
E130	T194	-	0.82	-	-	-	-	-	-	0.37	-	-	1.00	0.38	-	-	-	-	-	-	-
E130	R198	-	1.00	-	-	-	-	-	-	1.00	-	-	1.00	1.00	1.00	-	-	-	1.00	-	1.00
V132	F137	-	-	-	-	-	-	-	-	0.73	-	-	-	-	-	-	-	-	-	-	0.22
T133	F137	-	0.95	-	-	-	-	-	-	-	-	-	0.97	0.97	0.47	-	-	-	0.99	-	-
T133	R198	-	0.28	-	-	-	-	-	-	0.56	-	-	-	-	-	-	-	-	-	-	-
V136	E140	0.97	0.91	0.28	-	-	-	-	-	-	-	-	-	0.58	0.15	-	-	-	0.90	-	0.53
F137	Y141	0.99	0.85	0.66	-	-	-	-	-	0.17	-	-	0.98	0.98	0.17	-	-	-	0.72	-	-
E140	Y95	0.98	-	0.93	-	-	-	-	-	0.98	-	-	-	0.55	0.12	-	-	-	0.31	-	1.00
E140	R144	1.00	1.00	1.00	-	-	-	-	-	1.00	-	-	1.00	0.55	1.00	-	-	-	1.00	-	0.87
E140	R201	1.00	1.00	1.00	-	-	-	-	-	1.00	-	-	1.00	1.00	1.00	-	-	-	1.00	-	1.00
Y141	Q204	-	-	-	0.75	-	-	-	-	-	-	-	-	0.27	-	-	0.39	0.20	-	-	-
T194	R198	-	-	-	-	-	-	-	1.00	0.94	-	-	-	-	-	-	0.84	-	1.00	-	-
R201	Q204	1.00	-	0.44	-	0.84	0.61	0.25	-	1.00	-	0.32	1.00	0.28	-	0.85	0.67	0.33	0.26	-	-
R201	I205	-	-	0.95	-	-	0.78	0.35	-	0.53	-	0.51	-	0.82	-	-	0.43	0.76	0.64	-	-
L203	R207	0.36	-	0.73	-	0.39	0.87	-	-	0.81	-	0.70	0.51	0.31	-	0.44	0.38	0.74	0.17	-	-
Q204	R207	-	1.00	0.16	-	0.51	0.52	0.80	-	-	-	0.80	-	-	-	0.57	-	-	-	1.00	-
Q204	M208	-	0.41	0.83	-	0.73	0.89	-	-	-	-	0.56	-	-	-	0.59	0.72	0.85	-	0.88	-
L206	R210	-	0.80	0.85	-	-	0.88	0.95	-	-	-	0.64	-	-	-	0.79	-	0.95	-	0.80	-
R207	M211	-	-	-	-	-	-	-	-	-	-	-	-	-	-	-	-	-	-	-	-
# Activated																					
# Resting		3	3	2		1	2	1		3		1	3	2	2	2	1		2	1	2

BS-RTG		4																			
SIMULATION		1				2				3				4				5			
VSD domain		1	2	3	4	1	2	3	4	1	2	3	4	1	2	3	4	1	2	3	4
Y82	E86	1.00	0.86	0.75	0.23	0.13	0.91	0.93	0.80	0.61	0.90	0.83	0.13	0.60	0.91	0.90	-	1.00	0.87	0.86	0.38
E86	R87	-	0.80	-	-	-	1.00	-	-	0.53	1.00	-	-	-	1.00	-	-	-	1.00	-	-
E86	R144	-	1.00	-	-	-	0.29	-	-	0.81	-	0.62	-	-	0.42	0.58	-	-	0.33	0.54	-
E86	R207	-	1.00	-	-	-	1.00	1.00	-	-	1.00	1.00	-	-	1.00	1.00	-	-	1.00	1.00	-
E130	I134	0.58	0.95	-	-	-	-	0.56	-	-	-	-	-	0.89	0.67	0.95	-	0.95	-	-	-
E130	S179	0.98	-	-	-	-	-	0.84	-	-	-	-	-	1.00	1.00	0.91	-	-	-	-	-
E130	T194	0.97	-	-	-	-	-	0.96	-	-	-	-	-	-	1.00	0.40	-	-	-	-	-
E130	R198	1.00	1.00	-	-	-	-	1.00	-	-	-	-	-	0.94	1.00	1.00	-	1.00	-	-	-
V132	F137	0.75	-	-	-	-	-	-	-	-	-	-	-	0.56	0.83	-	-	0.90	-	-	-
T133	F137	-	0.96	-	-	-	-	0.76	-	-	-	-	-	-	-	0.99	-	-	-	-	-
T133	R198	0.89	-	-	-	-	-	-	-	-	-	-	-	-	0.48	-	-	-	-	-	-
V136	E140	0.92	0.95	-	-	0.85	-	-	-	-	-	-	-	0.89	0.73	0.24	-	-	-	-	-
F137	Y141	0.93	0.79	-	-	0.98	-	-	-	-	-	-	-	0.97	0.92	0.98	-	-	-	-	-
E140	Y95	0.89	0.94	-	-	0.99	-	-	-	0.72	-	-	-	0.99	0.76	0.89	-	-	-	-	-
E140	R144	0.72	1.00	-	-	1.00	-	1.00	-	0.79	-	-	-	0.78	0.97	1.00	-	-	-	-	-
E140	R201	0.91	1.00	-	-	1.00	-	1.00	-	1.00	-	-	-	1.00	1.00	1.00	-	-	-	-	-
Y141	Q204	0.39	-	-	0.78	-	-	-	-	0.12	-	-	-	0.16	-	-	0.57	0.12	-	-	1.00
T194	R198	0.88	-	-	-	-	-	-	-	-	0.92	0.96	0.98	0.89	-	-	-	0.45	-	-	-
R201	Q204	0.21	0.22	0.44	0.13	0.49	-	0.55	1.00	-	-	0.41	1.00	1.00	-	1.00	-	-	-	1.00	1.00
R201	I205	0.90	0.95	0.53	0.69	0.57	-	0.98	-	0.64	-	0.87	0.76	0.57	0.93	-	0.83	-	0.93	-	0.85
L203	R207	0.38	0.72	0.35	-	0.17	-	0.77	0.25	-	-	-	0.79	0.77	0.89	0.26	0.66	-	0.71	-	0.73
Q204	R207	-	0.40	0.37	-	0.75	1.00	-	0.36	-	-	0.63	-	-	0.45	0.77	-	-	0.54	-	-
Q204	M208	-	0.81	0.19	-	0.27	0.92	-	0.46	-	-	-	-	0.93	0.90	0.61	-	-	0.78	-	-
L206	R210	-	0.99	0.91	-	0.89	0.98	-	0.79	-	-	0.47	-	0.75	0.91	0.43	-	-	0.11	-	-
R207	M211	-	-	-	-	0.70	-	-	0.37	-	-	0.38	-	-	-	-	-	-	-	-	-
# Activated																					
# Resting		2	3			1	1	4	1	1	1	1	1	3	3	4		1	1	1	1

Table S7. List of interactions recorded within the VSD domains along different MD simulations of the complex between one RTG molecule and the KCNQ2 channel in the RC conformational state. See the caption of Table S2 for further details.

Supplementary references

Li X, Zhang Q, Guo P, et al (2021) Molecular basis for ligand activation of the human KCNQ2 channel. Cell Res 31:52–61. https://doi.org/10.1038/s41422-020-00410-8
